# MGST1 facilitates novel KRAS^G12D^ inhibitor resistance in KRAS^G12D^-mutated pancreatic ductal adenocarcinoma by inhibiting ferroptosis

**DOI:** 10.1186/s10020-024-00972-y

**Published:** 2024-11-05

**Authors:** Chungui Xu, Weihao Lin, Qi Zhang, Yarui Ma, Xue Wang, Ai Guo, Guiling Zhu, Zhendiao Zhou, Weiwei Song, Ziyi Zhao, Yuchen Jiao, Xiaobing Wang, Chunxia Du

**Affiliations:** 1 State Key Laboratory of Molecular Oncology, National Cancer Center/National Clinical Research Center for Cancer/Cancer Hospital, Chinese Academy of Medical Sciences and Peking Union Medical College, Beijing, 100021 China; 2 Institute of Cancer Research, Henan Academy of Innovations in Medical Science, Zhengzhou, Henan, 450000 China; 3 Department of Thoracic Surgery, National Cancer Center/National Clinical Research Center for Cancer/Cancer Hospital, Chinese Academy of Medical Sciences and Peking Union Medical College, Beijing, 100021 China; 4 Harrow international School Shenzhen Qianhai, Shenzhen, 518000 China; 5 Department of Medical Oncology, National Cancer Center/National Clinical Research Center for Cancer/Cancer Hospital, Chinese Academy of Medical Sciences and Peking Union Medical College, Beijing, 100021 China

**Keywords:** MGST1, Ferroptosis, Lipid peroxidation, MRTX1133 resistance, Pancreatic cancer

## Abstract

**Background:**

Pancreatic ductal adenocarcinoma (PDAC) is a highly lethal cancer with a low 5-year survival rate. Treatment options for PDAC patients are limited. Recent studies have shown promising results with MRTX1133, a KRAS^G12D^ inhibitor that demonstrated potent antitumor activity in various types of tumors with KRAS^G12D^ mutation. Resistance to KRAS inhibitors is frequently occurred and one of the main reasons for treatment failure. Understanding resistance mechanisms to novel KRAS inhibitors is crucial to ensure sustained and durable remissions.

**Methods:**

Two KRAS^G12D^ inhibitor MRTX1133-resistant PDAC cell lines were established in vitro. The resistance mechanisms to KRAS^G12D^ inhibitor MRTX1133 against PDAC in vitro and in vivo were characterized by RNA sequencing, reverse transcript polymerase chain reaction, cytotoxicity test, plasmid transfection, lentivirus transfection, lipid peroxidation detection, malondialdehyde levels detection, glutathione levels detection, western blot, immunofluorescence, nude mice tumorigenesis experiment and immunohistochemistry.

**Results:**

The bioinformatics analysis and transcriptome sequencing showed that ferroptosis was involved in the resistant effect of the KRAS^G12D^ inhibitor treatment, and MGST1 was the key molecule against MRTX1133-induced ferroptosis. Increased expression of MGST1 weakened the cytotoxicity of MRTX1133 by inhibiting lipid peroxidation-induced ferroptosis in KRAS^G12D^ inhibitor-resistant PDAC cells. Knockdown or overexpression of MGST1 conferred sensitivity or resistance to KRAS^G12D^ inhibitor MRTX1133, respectively. Mechanismly, increased nuclear localization and higher levels of active β-catenin were observed in MRTX1133-resistant PDAC cells, which contributed to higher MGST1 expression. Knockdown of CTNNB1 or TCF4 can decreased MGST1 expression. Additionally, we found that PKF-118-310, an antagonist of β-catenin/Tcf4 complex, repressed MGST1 expression. In both in vitro and in vivo models, a synergistic effect was observed when combining MRTX1133 and PKF-118-310 in KRAS^G12D^ inhibitor MRTX1133-resistant PDAC cells and tumors.

**Conclusion:**

Our data showed that KRAS^G12D^ inhibitor MRTX1133 combined with PKF-118-310 could enhance the effectiveness of MRTX1133 treatment response through induction of ferroptosis via inhibiting MGST1 expression in MRTX1133-resistant PDAC cells and tumors. This evidence may provide a promising strategy to overcome KRAS^G12D^ inhibitor MRTX1133 resistance in PDAC patients with KRAS^G12D^ mutations.

**Graphical Abstract:**

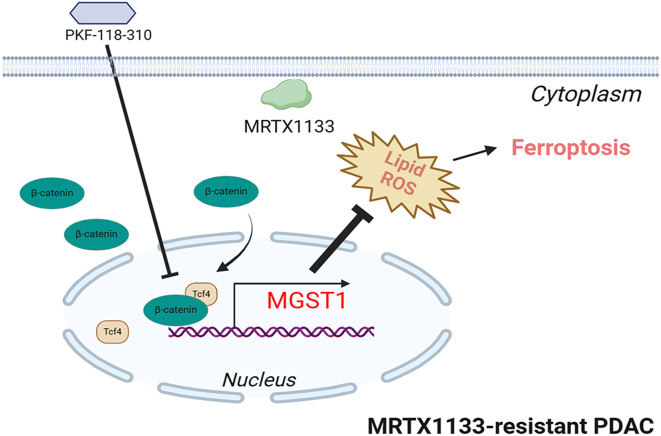

**Supplementary Information:**

The online version contains supplementary material available at 10.1186/s10020-024-00972-y.

## Introduction

Pancreatic ductal adenocarcinoma (PDAC) is a highly lethal cancer, with a 5-year survival rate of only 11% (Siegel et al. 2022). It is the third leading cause of cancer-related death in the United States (Park et al. 2021) and the seventh globally (Sung et al. 2021). The majority of patients are diagnosed in an advanced stage, with more than 80% of them having unresectable tumors and showing broad tolerance to chemoradiotherapy and immunotherapy. Consequently, these treatment outcomes are generally unfavorable (Hosein et al. 2022).

Kirsten rat sarcoma viral oncogene homolog (KRAS) is a frequently mutated gene in various types of solid tumors, such as colorectal cancer, lung adenocarcinoma, and PDAC (Li et al. 2018). The majority of PDAC patients, over 90%, carry oncogenic mutations in the KRAS gene (Hosein et al. 2022), making it an appealing target for new PDAC treatments. Previous attempts to target KRAS were unsuccessful until the recent development of KRAS^G12C^ inhibitors like Adagrasib (MRTX849) and Sotorasib (AMG510). These inhibitors have now been approved by the FDA (Huang et al. 2021; Ou et al. 2022). However, the KRAS^G12C^ mutation is only present in about 1% of PDAC patients, limiting the potential utility of these inhibitors (Hosein et al. 2022).

Notably, approximately 35% of PDAC patients harbor the KRAS^G12D^ mutation (Hosein et al. 2022), highlighting the importance of this specific genetic variant. A recent study has shown promising preclinical results with a potent, selective, and non-covalent high-affinity KRAS^G12D^ inhibitor named MRTX1133 (Mirati Therapeutics, San Diego, CA). This inhibitor has shown promising results in treated KRAS^G12D^ mutated colon, lung and PDAC, as indicated by previous studies (Wang et al. 2022a; Hallin et al. 2022). Treatment with MRTX1133 in mice models with implanted and autochthonous KRAS^G12D^ mutated tumours resulted in tumour regression and further led to notable alterations in the tumour immune microenvironment (TME). These changes included a decrease in tumour-infiltrating myeloid-derived suppressor cells (MDSCs), an increase in M1-like macrophages, and enhanced cytotoxic T cell activity (Kemp et al. 2023). Additionally, there is an ongoing clinical trial (NCT05737706) focusing on patients with advanced solid tumours carrying the KRAS^G12D^ mutation.

Inhibiting KRAS^G12C^ in colorectal cancer and non-small cell lung cancer resulted in a relatively short response due to the rapid development of resistance mechanisms, including both genetic and non-genetic factors (Awad et al. 2021). Consequently, the clinical efficacy of KRAS^G12D^ inhibitors, such as MRTX1133, may also be constrained by resistance. Nevertheless, the aforementioned trials did not evaluate the effect of acquired resistance on the efficacy of MRTX1133.

Ferroptosis is a newly discovered type of cell death that occurs when iron accumulates and leads to the production of lipid reactive oxygen species (ROS). This process finally causes the plasma membrane to rupture and expel the contents of the cells (Ursini and Maiorino 2020). Multiple studies have demonstrated that ferroptosis plays crucial roles in different types of malignancies, including its ability to reverse resistance to drug therapies, targeted therapies, and immunotherapies (Zhang et al. 2022; Chen et al. 2021). Intriguingly, tumours with KRAS mutations are especially susceptible to ferroptosis. The presence of constantly activated KRAS mutations directs the rearrangement of lipid metabolism and promote the generation of the excessive amount of ROS in cancer cells. This renders the cells highly responsive to alterations in metabolic processes (Mukhopadhyay et al. 2021). Consistent with the established connections between KRAS and NRF2, it has been demonstrated that restricting glutamine can trigger pro-ferroptosis signals, such as GPX4 suppression, in pancreatic cancer cells with KRAS mutations (Mukhopadhyay et al. 2020). These findings indicate that cancer cells with KRAS mutations and increased glutaminolysis may exhibit heightened susceptibility to ferroptosis.

Microsomal glutathione S-transferase 1 (MGST1) is an enzyme located on the cell membrane that facilitates the transfer of glutathione and performs glutathione peroxidase activities. It plays a crucial role in protecting cells from harmful or reactive substances (Kelner et al. 2000; Morgenstern et al. 2011). According to these findings, MGST1 has a significant impact on cancer cells and could be a promising target for therapy. Elevated MGST1 expression has been observed in malignant and drug-resistant melanomas. Targeting MGST1 has shown to alter the redox balance, limit metastases, and enhance the efficacy of chemo- and immunotherapies in melanoma (Zhang et al. 2023). MGST1 production has been found to inhibit ferroptosis by suppressing lipid peroxidation, although it does not impede iron accumulation in PDAC cells (Kuang et al. 2021). The relationship between MGST1 expression, ferroptosis inhibition, and resistance against KRAS^G12D^ inhibitor in PDAC remains uncertain.

In this study, our findings demonstrate that MGST1-mediated ferroptosis inhibition conferred the resistance of KRAS^G12D^ inhibitor MRTX1133 in PDAC. Suppression of MGST1 expression enhances the sensitivity to this inhibitor, while higher level of MGST1 expression has opposite effect. We also identified that the β-catenin/Tcf4 complex regulates the expression of MGST1. Treatment with PKF-118-310, an antagonist of the β-catenin/Tcf4 complex, led to a decrease in MGST1 expression in PDAC cells, thus resulting in increased susceptibility to the KRAS^G12D^ inhibitor MRTX1133. Furthermore, PKF-118-310 administration markedly enhanced the therapeutic efficacy of KRAS^G12D^ inhibitor MRTX1133 in both in vitro and in vivo models of MRTX1133-resistant PDAC cells and tumors. Taken together, our findings suggest that targeting MGST1 could be a promising strategy to overcome KRAS^G12D^ inhibitor MRTX1133 resistance in PDAC cells, with potential future benefits for patients with KRAS^G12D^-mutated PDAC.

## Methods and materials

### Cell lines and cell culture

The BxPC3, MIA PaCa-2, AsPC1, and 293T cell lines were purchased from the National Infrastructure of Cell Line Resource (Beijing, China). The KPC210 cell lines were isolated in our laboratory from a spontaneous *LSL-KRAS*^*G12D/+*^, *LSL-TRP53*^*R172H/+*^, *PDX1-CRE* (KPC) tumor following previously established protocols (Kamerkar et al. 2017; Zheng et al. 2015). BxPC3, MIA PaCa-2, 293T and KPC210 cells were cultured in Dulbecco’s modified Eagle’s medium (DMEM) supplemented with 10% fetal bovine serum (FBS) and 1% antibiotic mixture containing 100 U/mL penicillin and 100 mg/mL streptomycin (P/S). AsPC1 cells were cultured in Roswell Park Memorial Institute (RPMI)-1640 medium, supplemented with 10% FBS and 1% P/S. The cell lines were grown at a temperature of 37 °C in a 5% CO2 humidified environment. All experiments were conducted using mycoplasma-free cells.

### Bioinformatics analysis

The dataset GSE228502 was obtained from the GEO database and analyzed to identify the critical genes that exhibited resistance to MRTX1133 therapy in MRTX1133-treated KPC cells or ductal cells of C57BL6/J mice following MRTX1133 treatment. The KPC cells that were not treated with MRTX1133 named untreated KPC cells in our study, including cells utilized as control and cells treated with DMSO. Differential expression analysis was performed based on a *t*-test and fold change (FC). The Kyoto Encyclopedia of Genes and Genomes (KEGG) signaling pathway enrichment analysis were performed by using Metascape (Zhou et al. 2019). Protein-protein interaction (PPI) network of higher expressed genes was constructed. This network consisted of 74 nodes and 238 edges. In addition, the degree of node connections was determined using the Analyze Network function. 7 hub genes were identified with connectivity degrees exceeding 9.

### Reagents

MRTX1133, Erastin, Deferoxamine mesylate, Ferrostatin-1, and PKF-118-310 were purchased from MedChemExpress (Shanghai, China). Each of these agents was prepared as a 5mM stock solution dissolved in dimethylsulfoxide (DMSO) for cell experiment. Each was stored at -20 °C, and thawed and diluted with complete medium before each experiment. The ultimate concentration of DMSO did not exceed 0.1%.

### Establishment of the MRTX1133-resistant PDAC cells

AsPC1 cells and KPC210 cells were exposed to 1 nM-1 µM MRTX1133 for 24–48 h, depending on their viability. The cells were cultured in a medium without MRTX1133 until the viable cells had regained their usual exponential growth rate. The concentration of MRTX1133 was gradually raised from 1 nM to 1 µM. Following a period of 6 months, both the AsPC1 cells and KPC210 cells exhibited resistance to 1 µM MRTX1133. The newly established MRTX1133-resistant AsPC1 cells were named AsPC1-MR cells, and MRTX1133-resistant KPC210 cells were named KPC210-MR cells. Throughout the formation of AsPC1-MR cells and KPC210-MR cells, their parental cells were consistently cultured in a media devoid of MRTX1133 simultaneously. After being incubated in MRTX1133-free medium for almost 1 month, both the AsPC1-MR cells and KPC210-MR cells maintained their resistance to MRTX1133, indicating their stability.

### RNA sequencing

Parental AsPC1 cells, AsPC1 cells treated with 1 µM MRTX1133 for 48 h (AsPC1-D2), and AsPC1-MR cells were cultured in triplicate in 10 cm dishes. Total RNA was extracted using the RNA-quick purification kit (Yishan Biology Technology, Shanghai, China) following the manufacturer’s instructions. Illumina (San Diego, CA) Next-seq 5000 was used to assess the library quality, followed by data acquisition on the Illumina Hi-seq 4000. The Gene set enrichment analysis (GSEA) were conducted with GSEA software (Subramanian et al. 2005).

### Reverse transcript polymerase chain reaction (RT-qPCR)

Total RNA was extracted from differently treated PDAC cells and nude mouse tumours using the RNA-quick purification kit (Yishan Biology Technology, Shanghai, China). The RT-qPCR analysis was conducted using the TB green premix Ex Taq Kit (Takara Biomedical Technology, Beijing, China). The relative expression levels of candidate genes were calculated using the 2^−ΔΔCT^ method. The RNA input for all RT-qPCR analyses was normalized using *GAPDH*. All primer sequences used in our study are listed in Table S1.

### Measurement of intracellular GSH, malondialdehyde (MDA), and lipid peroxidation

The intracellular GSH levels and MDA levels of cells treated with different reagents were measured using a commercial MDA assay kit (Solarbio Life Sciences, Beijing, China) and GSH assay kit (Beyotime Biotechnology, Shanghai, China), following the directions provided by the manufacturer. MDA in the samples reacted with thiobarbituric acid (TBA) to produce an MDA-TBA adduct, which can be conveniently measured using colorimetry at a wavelength of 532 nm. The levels of lipid ROS production were assessed using the BODIPY 581/591 C11 probe (Thermo Fisher Scientific, US) following the instructions provided by the manufacturer. A homogeneous solution of individual cells was produced from PDAC cells that had been subjected to various treatments, with a concentration of 1 × 10^6^ cells/mL. The cells were exposed to the probe at a concentration of 5 µM for 30 min at a temperature of 37 °C, and then washed three times with PBS. The data were obtained using an LSR-II instrument (BD, San Diego, CA) and analyzed using FlowJo software (Tree Star, Inc, Ashland, OR).

### RNAi and Plasmid transfection

RNA interference (RNAi) was used to perform the gene knockdown in AsPC1-MR cells. The cells were transfected with a concentration of 20 nM siRNA for 48 h. The human siRNAs used in this study were obtained from Sangon Biotech (Shanghai). The specific siRNAs used were MGST1-siRNA1 (sense: CGAACAGAUGACAGAGUAGAATT; antisense: UUCUACUCUGUCAUCUGUUCGTT), MGST1-siRNA2 (sense: CUUGGAAUUGGCCUCCUGUAUTT; antisense: AUACAGGAGGCCAAUUCCAAGTT), and SiNC (sense: UUC UCC GAA CGU GUC ACG UTT; antisense: ACG UGA CAC GUU CGG AGA ATT). The *MGST1* plasmid (OriGene, RC203251) was transfected into AsPC1 cells via transfection using a DNA transfection reagent (Neofect biotechnology, Beijing, China). The efficiency of the expression was assessed by western blot.

### Viral production and transduction

The LentiCRISPR v2 system was used to generate lentiviruses containing mouse MGST1 sgRNA (TAGAGAGATCTGGTCCACTC), human CTNNB1 sgRNA (AAGGTTATGCAAGGTCCCAG), human TCF4 sgRNA (CTAGCAATAATCCCCGAAGG), mouse CTNNB1 sgRNA (ATGAGCAGCGTCAAACTGCG), mouse TCF4 sgRNA (CAAGCAATAATGCCCGCCGG) for validation. The day before transfection, 4 × 10^7^ HEK293T cells were seeded into six-well plates to achieve at least 70% confluence. HEK293T cells were transfected with lentiCRISPR v2, psPAX2, and pMD2.G plasmids using Neofect transfection reagent (Neo Biotech, Beijing, China). After 60 h, the lentivirus containing medium were collected, combined, and cleared by centrifugation at 300 g for 10 min at 4 °C. AsPC1-MR cells or KPC210-MR cells were infected with the various lentiviral vectors. Subsequently, cells were selected with puromycin (2 µg/ml). Single colony was selected and the transfection efficiency was quantified by western blot.

### Western blot and immunofluorescence

The active β-catenin antibody was purchased from Cell Signaling Technology (Shanghai, China). The active β-catenin protein obtained from cells treated with various conditions was examined using SDS-PAGE (Beyotime Biotechnology, Shanghai, China). The western blot assay was conducted using the standard laboratory protocols. β-actin was used as the loading control. Signals were detected with ECL reagents (Applygen Technology, Beijing, China), and the images were captured using Amersham Imager 600 (GE Healthcare, USA). The levels of active β-catenin were quantified with ImageJ software. Immunofluorescence microscopy was conducted with standard laboratory protocols to assess the abundance of active β-catenin.

### Colony formation assay

AsPC1, AsPC1-MR, KPC210 and KPC210-MR cells were seeded into 96-well plated with different concentration of MRTX1133 (0 µM, and 1 µM) or PKF-118-310 (0 nM, 10 nM, 100nM, 200nM, and 500nM) per well. After treated with 3 days, the colonies were fixed in 10% formalin and stained with crystal violet.

### Drug sensitivity and drug synergy

To measure drug sensitivity, cells were plated in 96-well plates at a density of 5000 AsPC1 cells or AsPC1-MR cells, 3000 KPC210 cells or KPC210-MR cells per well, and allowed to adhere overnight. The cells were treated with various concentrations of the indicated drugs for three days. Cell viability was evaluated using Cell Counting Kit-8 assay (Meilunbio, Dalian, China), following the manufacturer’s instructions. The IC_50_ values in cultured cells were determined after the addition of different concentrations of MRTX1133 following the protocol of our previous report (Zhu et al. 2021). For synergy, a 1:1 drug combination was used. The CCK8 assay was conducted using different concentrations of MRTX1133 (ranging from 0 to 10 µM) and PKF-118-310 (ranging from 0 to 500 nM). The synergistic effects of the two drugs were assessed using Synergy Finder (Ianevski et al. 2020).

### Immunohistochemistry

Immunohistochemistry was conducted in paraffin-embedded tissues with standard laboratory protocols. The stained sections were scanned and analyzed using Aperio ScanScope software (Aperio Technologies, Vista, CA). Representative immunohistochemical images and statistical analysis of MGST1, Ki67, and 4-hydroxynonenal (4-HNE) staining from subcutaneous xenograft tissues in AsPC1-MR tumor-bearing model. A positive score of MGST1 and 4-HNE in tumor samples was quantified based on the percentage area and intensity of positive staining, and the numbers of Ki67^+^ tumor cells in each section from 3 independent fields under 200× as reported (Huang et al. 1997; Qu et al. 2018).

### Animals

Nude mice aged 6 weeks and weighing 18–25 g were purchased from Huafukang Biological Technology (Beijing, China). In order to monitor the progression of tumours at various time intervals, a total of 1 × 10^6^ AsPC1-MR cells, 3 × 10^5^ KPC210-MR cells, or 3 × 10^5^ MGST1 KO cells were subcutaneously injected into the left flank of nude mice. Upon tumour formation on Day 3, the mice received intraperitoneal injections of 1 mg/kg MRTX1133 (*n* = 3 or *n* = 4), intratumoral injections of 1 mg/kg PKF-118-310 (*n* = 3 or *n* = 4), a combination of 1 mg/kg MRTX1133 and 1 mg/kg PKF-118-310 (*n* = 3 or *n* = 4), or served as controls (*n* = 3 or *n* = 4). MRTX1133 was administered daily, while PKF-118-310 was given every three days (Wei et al. 2010).

Additionally, KPC210 cells, which were responsive to MRTX1133, were also injected into nude mice, who were then divided into four groups. When a tumour mass formed at Day 5, the mice were intraperitoneal injected with 3 mg/kg MRTX1133 (*n* = 4) (Hallin et al. 2022), intratumorally injected with 1 mg/kg PKF-118-310 (*n* = 4), a combination of 3 mg/kg MRTX1133 and 1 mg/kg PKF-118-310 (*n* = 4), or served as controls (*n* = 4).

The tumour size was measured every 2–3 days, and the tumour volume was calculated with the following formula: (length x width^2^)/2. N represents number of animals utilized in each group.

### Statistical analysis

All experiments were conducted independently at least thrice. Statistical analyses were performed using R version 4.0.2 (Vienna, Austria) or GraphPad Prism 8 (GraphPad, La Jolla, CA). Continuous variables were compared with an unpaired student *t*-test between 2 groups. The differences of discrete variables were compared using the Mann-Whitney test. Data were expressed as mean ± SEM. A *P* value less than 0.05 (2-tailed) was considered statistically significant.

## Results

### Bioinformatic analysis revealed that MGST1-mediated ferroptosis inhibition was observed in PDAC cells treated with KRAS^G12D^ inhibitor

**Fig. 1:**
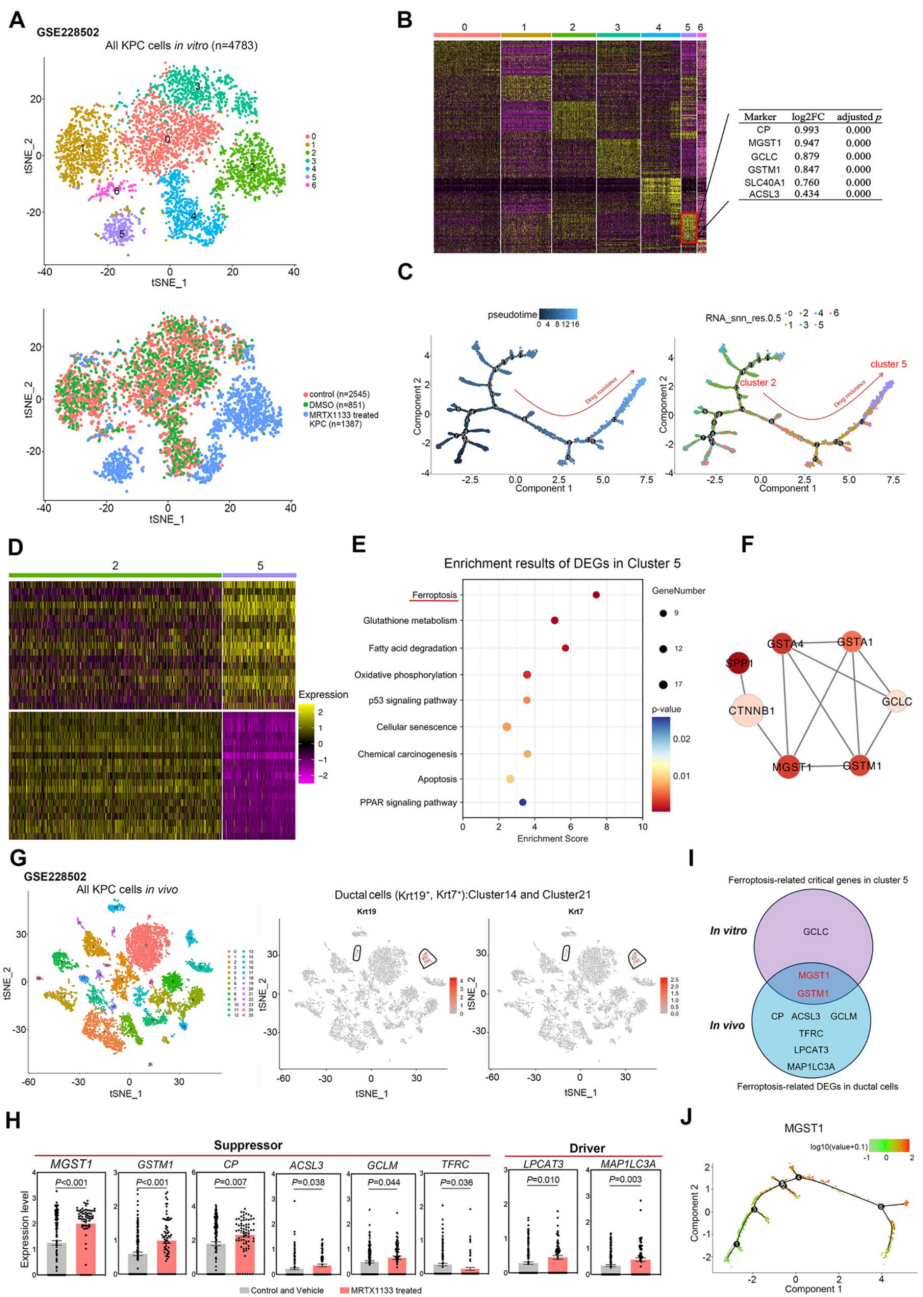
MGST1-mediated ferroptosis inhibition might contribute to the survival of MRTX1133-treated PDAC cells. (**A**) Unsupervised clustering of all KPC cells using the single-cell transcriptomes from GEO datasets GSE228502. (**B**) Distinct gene signatures of each cluster. Yellow and purple represent high and low expression, respectively. (**C**) The pseudo-time trajectories analysis of all KPC cells. (**D**) Difference analysis between KPC cells in Cluster 2 and Cluster 5. Yellow and purple represent high and low expression, respectively. (**E**) KEGG Enrichment analysis of significantly differentially expressed genes in Cluster 5 compared with Cluster 2. (**F**) 7 Hub genes identified by protein-protein interaction network analysis in Cluster 5. (**G**) Mouse ductal cells identified by the gene markers Krt19 and Krt7. (**H**) The significant expressed genes associated with ferroptosis in ductal cells of untreated mouse (Control and Vehicle) and of mouse treated with MRTX1133. (**I**) The Venn diagram showing 2 overlapping genes associated with ferroptosis. (**J**) The pseudo-time trajectories analysis of MGST1 expression in Cluster 2 and Cluster 5. Data represent means ± SEM. *P* values were calculated by an unpaired student *t* test or the Mann–Whitney test. KEGG: Kyoto Encyclopedia of Genes and Genomes; PDAC: Pancreatic ductal adenocarcinoma; DEG: Differential expressed genes

Single cell transcriptomes of untreated KPC cells and KPC cells treated with 50 nM MRTX1133 were obtained from the GEO dataset GSE228502 (Mahadevan et al. 2023). A total of 3396 untreated KPC cells and 1387 MRTX1133-treated KPC cells were analyzed. We performed unsupervised clustering of all KPC cells and seven clusters were generated (Fig. 1A). Most cells in the Cluster 2 and Cluster 5 were the cells treated with MRTX1133, which reflected that there might be different biological features between untreated KPC cells and MRTX1133-treated KPC cells. Each cluster exhibited distinct signature genes, and we noticed that elevated expression of several genes associated with ferroptosis inhibition in the Cluster 5, including *CP*, *MGST1*, *GCLC*, *GSTM1*, *SLC40A1*, and *ACSL3* (Fig. 1B). Then, we utilized the R package “monocle” to compute and visualize the pseudo-time trajectories of all KPC cells. Cluster 5 was located near the final point of the trajectory (Fig. 1C). Compared with Cluster 2, the cells in Cluster 5 might be more easily to survival under KRAS^G12D^ inhibitor MRTX1133 treatment.

To further identify the critical genes for cell survival after MRTX1133 treatment, significantly different expressed genes (DEGs) were identified between the cells in Cluster 2 and the cells in Cluster 5 (Fig. 1D). The pathways of ferroptosis and glutathione metabolism were enriched in the Cluster 5 (Fig. 1E). Subsequently, we analyzed the genes with higher expression in Cluster 5 compared with Cluster 2 using the STRING database platform for protein-protein interaction (PPI) network analysis. The results were then imported into Cytoscape software to construct the PPI network (Fig. S1A). Seven genes with a degree higher than 9 were identified as hub genes: *MGST1*, *GSTM1*, *CTNNB1*, *SPP1*, *GCLC*, *GSTA1*, and *GSTA4* (Fig. 1F).

We also obtained single-cell transcriptomes of pancreata/tumours from mice treated with or without KRAS^G12D^ inhibitor MRTX1133 from the GEO dataset GSE228502 for subsequent analysis (Mahadevan et al. 2023). Unsupervised clustering revealed that cells in Cluster 14 and Cluster 21 were ductal cells (Fig. 1G). Ductal cells from untreated mice and those treated with MRTX1133 showed distinct differences in genes associated with the ferroptosis pathway (Fig. S1B). Six genes associated with ferroptosis inhibition were significantly higher expressed in the ductal cells from mice treated with MRTX1133, including *MGST1*, *GSTM1* and *CP* (Fig. 1H). The ferroptosis suppressors *MGST1* and *GSTM1* were higher expressed under MRTX1133 treatment in both in vitro and in vivo models (Fig. 1I). Also, the expression levels of *MGST1* significantly increased towards the end of the trajectory compared to the beginning (Fig. 1J and S1C). Above results demonstrated that MGST1 expression was enhanced in PDAC cells or tumors, which might prevent ferroptosis and be more easily to survival under KRAS^G12D^ inhibitor MRTX1133 treatment. Thus, we focused on ferroptosis to further study potential mechanisms of MRTX1133 resistance in PDAC.

### KRAS^G12D^ inhibitor MRTX1133 induces ferroptosis in PDAC cells

**Fig. 2:**
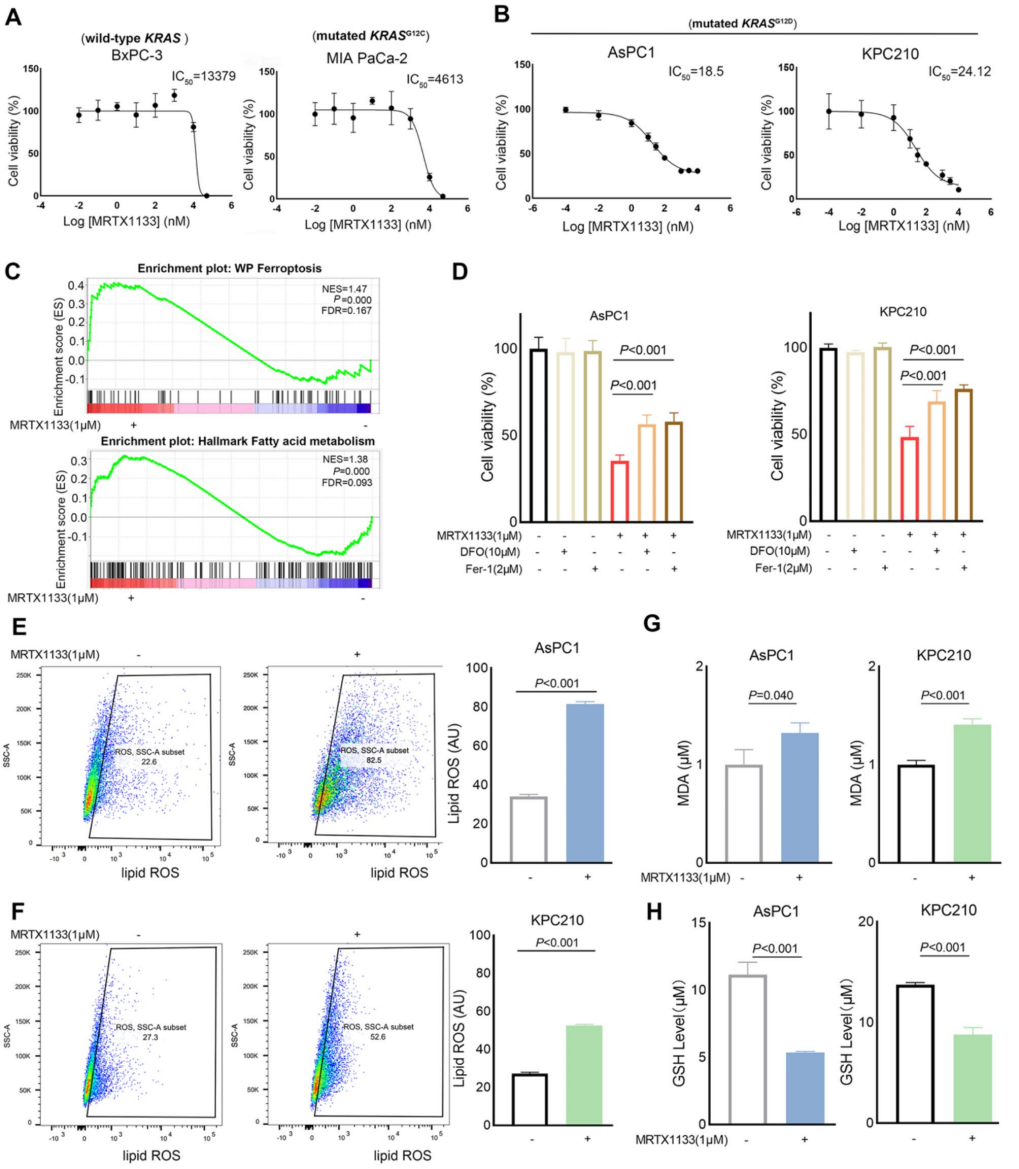
KRAS^G12D^ inhibitor MRTX1133 induced ferroptosis in PDAC cells. (**A**) The IC_50_ of MRTX1133 treated for 72 h in BxPC3 and MIA PaCa-2 cells. (**B**) The IC_50_ of MRTX1133 treated for 72 h in AsPC1 and KPC210 cells. (**C**) GSEA analysis showed the enrichment of ferroptosis and fatty acid metabolism in the AsPC1 cells treated with 1 µM MRTX1133 for 48 h. (**D**) The effects of DFO and Fer-1 on cell viability after MRTX1133 treatment were assessed by CCK8 assay. Pretreatment with DFO (10 µM) or Fer-1 (2 µM) for 4 h and the other indicated drugs was carried out for 48 h. (**E**, **F**) The lipid peroxidation was assessed by BODIPY 581/591 C11 probe using a flow cytometer after the 1 µM MRTX1133 treatment for 48 h. (**G**) The intracellular MDA level was assessed after the 1 µM MRTX1133 treatment for 48 h. (**H**) The intracellular GSH level was assessed after the 1 µM MRTX1133 treatment for 48 h. Data represent means ± SEM from three independent experiments. *P* values were calculated by an unpaired student *t* test. IC_50_: Half maximal inhibitory concentration; GSEA: Gene set enrichment analysis; DFO: deferoxamine mesylate; Fer-1: ferrostatin-1; ROS: Reactive oxygen species; MDA: malondialdehyde; GSH: Glutathione

To examine the anti-tumor potential of the KRAS^G12D^ inhibitor MRTX1133 on PDAC cells, we firstly measured the half-maximal inhibitory concentration (IC_50_). The BxPC-3 cells, with wild type KRAS, and the MIA PaCa-2 cells, which harbors KRAS^G12C^ mutation, exhibited comparative resistance to MRTX1133. The IC_50_ of MRTX1133 was 13,379 nM in BxPC-3 cell lines and 4613 nM in MIA PaCa-2 cell lines (Fig. 2A). Nevertheless, the human PDAC cell line AsPC1 and the mouse PDAC cell line KPC210, both with KRAS^G12D^ mutations, exhibited sensitivity to MRTX1133. The IC_50_ of MRTX1133 was 18.5 nM in AsPC1 cells, and 24.12 nM in KPC210 cells (Fig. 2B). These results confirmed that anti-tumor effect of MRTX1133 on tumors with KRAS^G12D^ mutations and were consistent with prior research (Wang et al. 2022a; Hallin et al. 2022).

Following RNA sequencing (RNA-seq) of parental AsPC1 cells and AsPC1 cells treated with 1 µM MRTX1133, GSEA results revealed a distinct enrichment of gene signatures, including “ferroptosis” and “fatty acid metabolism” (Fig. 2C). To further confirm the role of ferroptosis in MRTX1133 treated PDAC cells, AsPC1 and KPC210 cells were treated with MRTX1133 in the absence or presence of two ferroptosis inhibitors, and we found that treatment combined with deferoxamine mesylate (DFO) or ferrostatin-1 (Fer-1) partly offset the cytotoxic effect under KRAS^G12D^ inhibitor MRTX1133 treatment (Fig. 2D). One of the characteristics of ferroptosis is the accumulation of phospholipid hydroperoxide products (D’Herde and Krysko 2017). The BODIPY 581/591 C11 probe was used to detect the lipid peroxidation levels. Results showed that the lipid peroxidation levels in AsPC1 and KPC210 cells were significantly increased under MRTX1133 treatment (Fig. 2E and F). The oxidative stress marker MDA and GSH depletion are also the important events in ferroptosis (Koppula et al. 2021; Kuang et al. 2020; Chen et al. 2020). Along with MDA accumulation (Fig. 2G), depletion of GSH was observed in both AsPC1 and KPC210 cells (Fig. 2H). These results indicated that the KRAS^G12D^ inhibitor MRTX1133 treatment could induce ferroptosis in PDAC.

### Ferroptosis inhibition and enhanced MGST1 expression conferred the KRAS^G12D^ inhibitor MRTX1133 resistance in PDAC

**Fig. 3:**
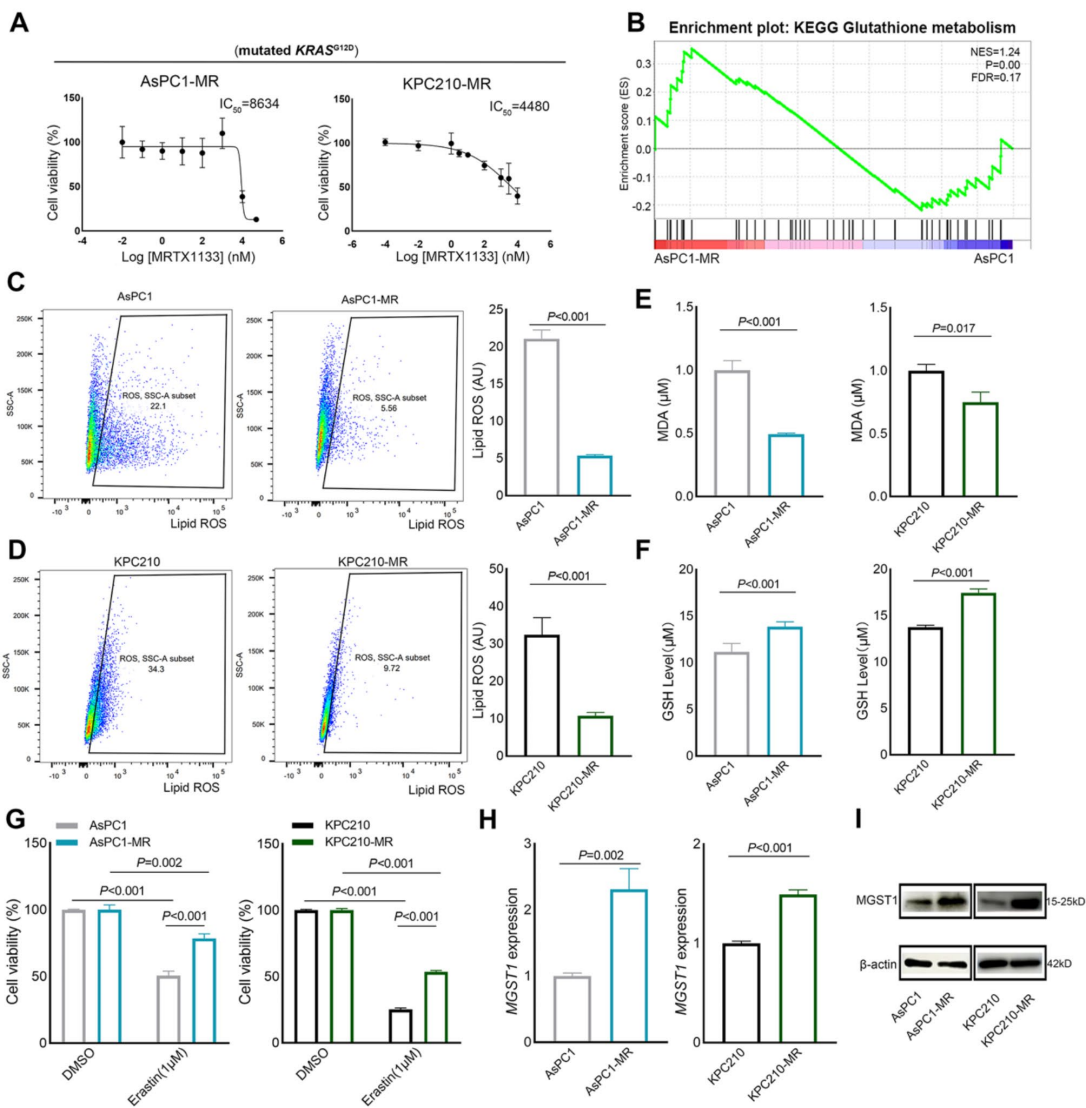
Ferroptosis inhibition and upregulation of MGST1 expression conferred the KRAS^G12D^ inhibitor MRTX1133 resistance in PDAC cells. (**A**) The IC_50_ of MRTX1133 treated for 72 h in MRTX1133-resistant PDAC cells. (**B**) GSEA analysis showed the enrichment of glutathione metabolism in the AsPC1-MR cells. (**C**,** D**) The lipid peroxidation was assessed by BODIPY 581/591 C11 probe using a flow cytometer in parental PDAC cells and MRTX1133-resistant PDAC cells. (**E**) The intracellular MDA level was assessed in parental PDAC cells and MRTX1133-resistant PDAC cells. (**F**) The intracellular GSH level was assessed in parental PDAC cells and MRTX1133-resistant PDAC cells. (**G**) The cell viability was measured after 1 µM Erastin treatment were assessed by CCK8 assay in parental PDAC cells and MRTX1133-resistant PDAC cells. (**H**) The expression levels of MGST1 in parental PDAC cells and MRTX1133-resistant PDAC cells. (**I**) The protein levels of MGST1 in parental PDAC cells and MRTX1133-resistant PDAC cells. Representative images were shown here and other images were represented in Supplementary Fig. 5A. Data represent means ± SEM from three independent experiments. *P* values were calculated by an unpaired student *t* test. IC_50_: Half maximal inhibitory concentration; GSEA: Gene set enrichment analysis; ROS: Reactive oxygen species; MDA: malondialdehyde; GSH: Glutathione

Following a 6-months treatment with increasing concentrations (1 nM − 1 µM) of KRAS^G12D^ inhibitor MRTX1133, two MRTX1133-resistant PDAC cell lines were established, named AsPC1-MR cells and KPC210-MR cells (Fig. S2A). The IC_50_ of MRTX1133 was 8634 nM in AsPC1-MR cells and 4480 nM in KPC210-MR cells (Fig. 3A). Compared with parental PDAC cells, MRTX1133-resistant PDAC cells weakened the cytotoxicity of the MRTX1133 treatment (Fig. S2B). Colony formation assays also indicated AsPC1-MR and KPC210-MR cells were more resistant to MRTX1133 (Fig. S2C). RNA-seq was performed between AsPC1 cells and AsPC1-MR cells. We then conducted gene set enrichment analysis (GSEA) to understand transcriptional difference in parental cells and MRTX1133-resistant cells. AsPC1-MR cells showed different biological capabilities from AsPC1 cells (Table. S2). The high expressed genes in MRTX1133-resistant cells were associated with Notch signaling, angiogenesis, and regulation of Hippo signaling (Fig. S2D). Notably, result showed that the glutathione metabolism pathway was enriched in AsPC1-MR cells (Fig. 3B). The lipid peroxidation levels in AsPC1-MR and KPC210-MR cells were significantly decreased (Fig. 3C and D). The lower MDA levels and the higher GSH levels were also observed in AsPC1-MR and KPC210-MR cells compared to parental PDAC cells (Fig. 3E and F). Furthermore, we found that there were less fold change of MDA and GSH levels after MRTX1133 treatment in both AsPC1-MR and KPC210-MR cells compared to parental cells, respectively (Fig. S2E- S2F). Moreover, AsPC1-MR and KPC210-MR cells exhibited higher cell viabilities when treated with 1 µM Erastin, a ferroptosis-inducing compound (Fig. 3G). These findings identified that MRTX1133-resistant PDAC cells exhibit ferroptosis inhibition.

Then, we detected the expression levels of several genes associated with ferroptosis inhibition. RT-qPCR and western blot detection showed that the MGST1 expression was significantly higher in AsPC1-MR cells and KPC210-MR cells compared to parental cells, respectively (Fig. 3H and I). The expression levels of GSTM1 showed no difference (Fig. S2G). RT-qPCR analysis further confirmed elevated *MGST1* expression in AsPC1, KPC210, LS180, and A427 cells following treatment with MRTX1133 compared to parental cells (Fig. S2H- S2I). Additionally, the expression level of *MGST1* were significantly higher in the AMG510-resistant MIA PaCa2 cells compared to MIA PaCa2 cells (Fig. S2J). These results confirmed the results of bioinformatics analysis, and indicated that MGST1-mediated ferroptosis inhibition might confer the KRAS^G12D^ inhibitor MRTX1133 resistance.

### Alterations of MGST1-mediated ferroptosis inhibition promote KRAS^G12D^ inhibitor MRTX1133 induced ferroptosis

**Fig. 4:**
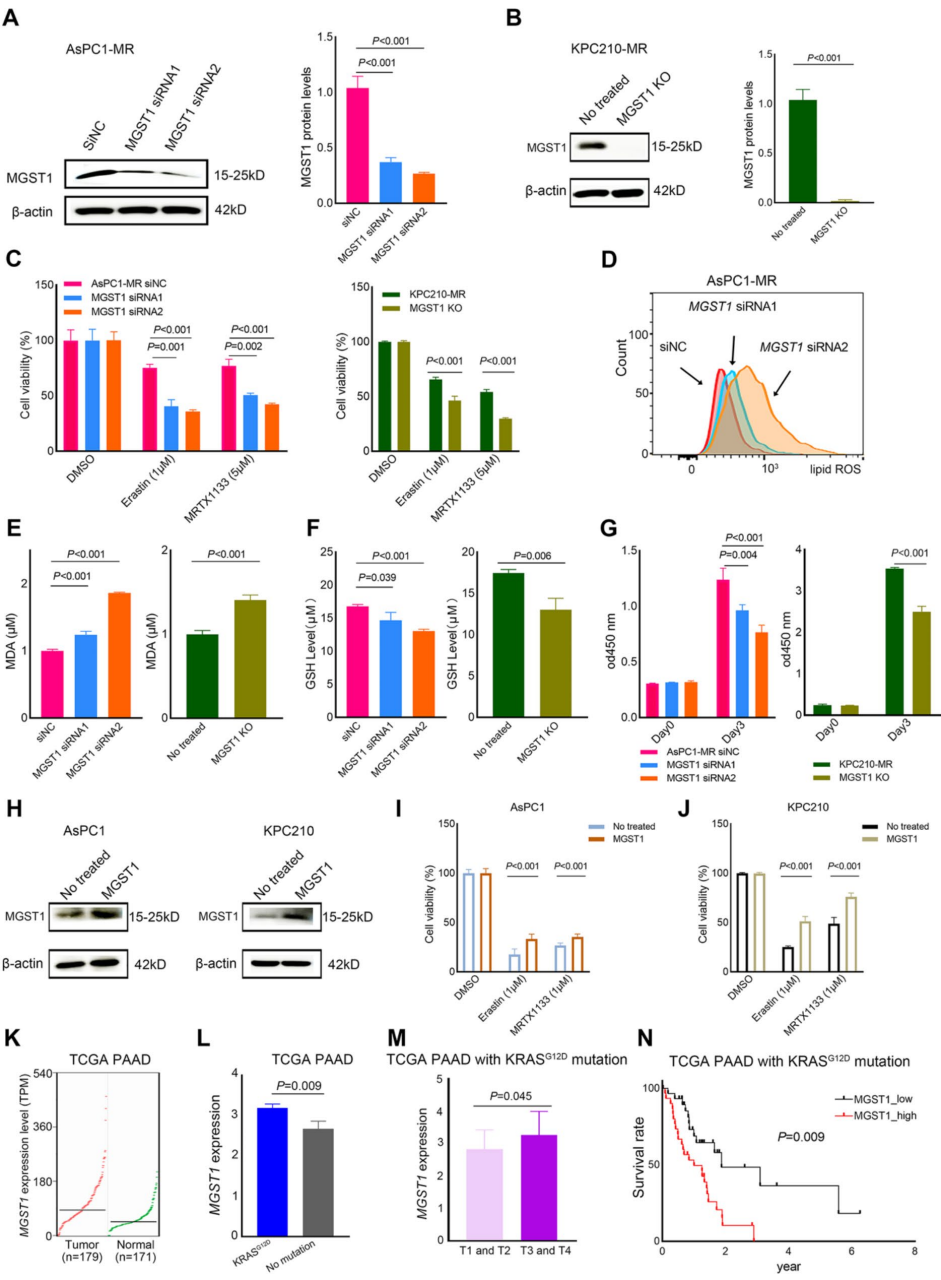
MGST1-mediated ferroptosis inhibition caused KRAS^G12D^ inhibitor MRTX1133 resistance. (**A**) The protein levels of MGST1 in AsPC1-MR cells transfected with different RNA interference (RNAi) molecules. AspC1-MR siNC was as the control cells. The protein levels were quantified using ImageJ software. Representative images were shown here and other images were represented in Supplementary Fig. 5B. (**B**) The protein levels of MGST1 in KPC210-MR cells subjected to the stable knockdown of MGST1 (MGST1 KO) was assessed by western blot. The protein levels were quantified using ImageJ software. Representative images were shown here and other images were represented in Supplementary Fig. 5C. (**C**) The cell viabilities of AsPC1-MR cells transfected with different RNAi molecules or KPC210-MR cells subjected to the stable knockdown of MGST1 treated with 1 µM Erastin or 5 µM MRTX1133 for 48 h. (**D**) The lipid peroxidation of AsPC1-MR cells transfected with different RNAi molecules assessed by flow cytometry after the treatment of 5 µM MRTX1133 for 48 h. (**E**) The intracellular MDA levels of MRTX1133-resistant PDAC cells subjected to knockdown of MGST1 was assessed after the treatment of 5 µM MRTX1133 for 48 h. (**F**) The intracellular GSH levels of MRTX1133-resistant PDAC cells subjected to knockdown of MGST1 was assessed after the treatment of 5 µM MRTX1133 for 48 h. (**G**) CCK8 assays were used to analyze the cell proliferation rate of MRTX1133-resistant PDAC cells subjected to knockdown of MGST1 for 72 h. (**H**) The protein levels of MGST1 in parental PDAC cells overexpressing MGST1. Representative images were shown here and other images were represented in Supplementary Fig. 5D. (**I**, **J**) The cell viabilities of parental PDAC cells overexpressing MGST1 treated with 1 µM Erastin or 1 µM MRTX1133 for 48 h. Data represent means ± SEM from three independent experiments. (**K**) The MGST1 mRNA expression levels in tumor group (*n* = 179) and normal samples (*n* = 171) of PDAC patients using TCGA databases. (**L**) The MGST1 mRNA expression levels in PDAC patients with KRAS^G12D^ mutation compared to PDAC patients with no mutations in KRAS. (**M**) The MGST1 mRNA expression levels in low-grade (T1 and T2) and high-grade (T3 and T4) of PDAC patients harbored with the KRAS^G12D^ mutation. (**N**) Kaplan-Meier overall survival analysis of MGST1 mRNA expression in PDAC patients harbored with the KRAS^G12D^ mutation. Data represent means ± SEM. *P* values were calculated by an unpaired student *t* test or the Mann–Whitney test. KO: knockout; ROS: Reactive oxygen species; MDA: malondialdehyde; GSH: Glutathione

Considering that MGST1 is an enzyme involved in the process of detoxification during oxidative stress and has been identified as a suppressor of ferroptosis (Morgenstern et al. 2011), we further examined the role of MGST1 in ferroptosis inhibition in MRTX1133-resistant PDAC cells. The expression of *MGST1* in AsPC1-MR cells was suppressed using two RNA interference molecules, siRNA1 and siRNA2 (Fig. 4A). And MGST1 was knockout by CRISPR-Cas9 in KPC210-MR cells (Fig. 4B). We observed that MGST1 knockdown decreased the viability of AsPC1-MR and KPC210-MR cells treated with Erastin or MRTX1133 (Fig. 4C). MGST1 knockdown decreased lipid ROS and MDA levels and increased GSH levels after MRTX1133 treatment in MRTX1133-resistant cells (Fig. 4D and F). We also observed that MGST1 knockdown decreased the cell proliferation rate (Fig. 4G). However, MGST1 overexpression in AsPC1 and KPC210 cells conferred resistance to Erastin and MRTX1133 (Fig. 4H and J). Overall, these results revealed that MGST1 decreased the sensitivity of PDAC cells to KRAS^G12D^ inhibitor MRTX1133 by ferroptosis inhibition.

We also used the TCGA database to analyze the MGST1 expression and clinical significance in PDAC patients with KRAS^G12D^ mutations. The expression level of MGST1 was upregulated in the tumor group (*n* = 179) compared with that in the normal group (*n* = 171) (Fig. 4K). Compared with patients without mutations in KRAS, the patients with KRAS^G12D^ mutations exhibited higher MGST1 expression (Fig. 4L). In PDAC patients with KRAS^G12D^ mutations, the expression level of MGST1 showed a substantial correlation with the pathological stage (Fig. 4M). The overall survival time for patients with high MGST1 mRNA expression were significantly shorter than those for patients with low MGST1 expression (Fig. 4N). Above results indicated that MGST1 could be a potential prognosis marker for poor outcomes, and higher MGST1 expression might cause MRTX1133 resistance in PDAC patients with KRAS^G12D^ mutations.

### MGST1 was regulated by β-catenin/Tcf4 complex in MRTX1133-resistant PDAC cells

**Fig. 5:**
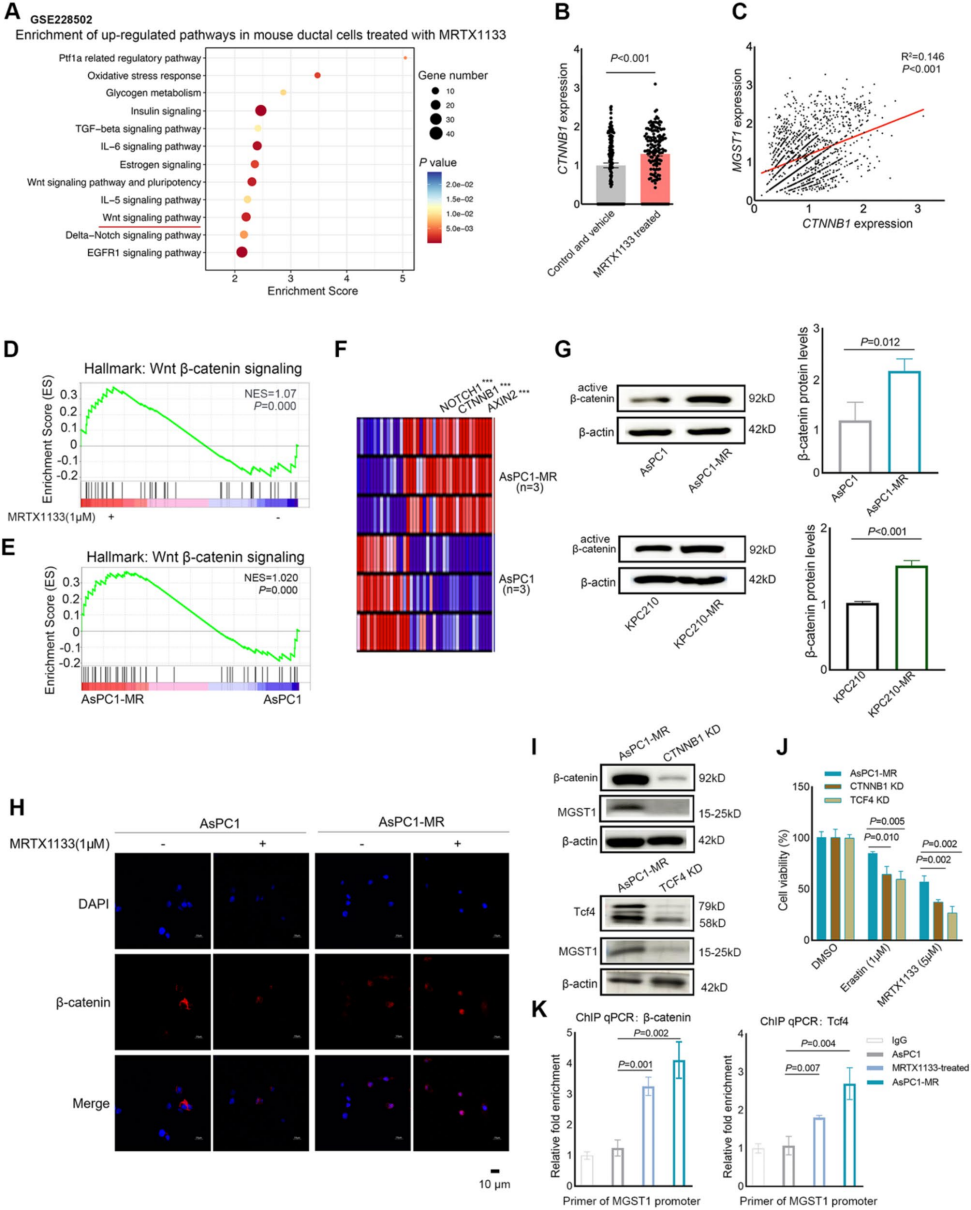
Upregulated MGST1 expression was modulated by the β-catenin/Tcf4 complex in MRTX1133-resistant PDAC cells. (**A**) KEGG enrichment analysis of higher expressed genes in ductal cells of mouse treated with MRTX1133. (**B**) The expression levels of CTNNB1 in ductal cells of untreated mouse (Control and Vehicle) and of mouse treated with MRTX1133. (**C**) A positive correlation between the CTNNB1 expression with MGST1 expression in mouse ductal cells. (**D**) GSEA analysis showed the enrichment of Wnt/β-catenin signaling in the AsPC1 cells treated with 1 µM MRTX1133 for 48 h. (**E**) GSEA analysis showed the enrichment of Wnt/β-catenin signaling in the AsPC1-MR cells compared with AsPC1 cells. (**F**) Heatmap of several critical gene expressions of the Wnt/β-catenin signaling pathway in AsPC1 cells and AsPC1-MR cells. (**G**) The active β-catenin protein levels in AsPC1 and AsPC1-MR cells were assessed by western blot. The protein levels were quantified using ImageJ software. Representative images were shown here and other images were represented in Supplementary Fig. 5A. (**H**) Immunofluorescence analysis of the β-catenin levels in the nucleus of MRTX1133-resistant cells and its parental cells treated with 1 µM MRTX1133 for 48 h. Scale bar: 10 μm. (**I**) The protein levels of MGST1 in AsPC1-MR cells subjected to the stable knockdown of CTNNB1 (CTNNB1 KO) or TCF4 (TCF4 KO) were assessed by western blot. Representative images were shown here and other images were represented in Supplementary Fig. 5E. (**J**) The cell viabilities of AsPC1-MR cells subjected to the stable knockdown of CTNNB1 or TCF4 treated with 1 µM Erastin or 5 µM MRTX1133 for 48 h. (**K**) The putative motif was more prominently detected in the DNA that was precipitated after the addition of the antibodies targeting β-catenin or Tcf4 in AsPC1-MR cells. Data represent means ± SEM from three independent experiments. *P* values were calculated by an unpaired student *t* test. KEGG: Kyoto Encyclopedia of Genes and Genomes; GSEA: Gene set enrichment analysis

The transcription factor of NFE2L2 is recognized as upstream regulatory of MGST1 in PDAC (Kuang et al. 2021), but AsPC1-MR cells showed decreased NFE2L2 expression compared with AsPC1 cells (Fig. S3A). Thus, we further explore to reveal the potential mechanism of upregulation of MGST1. Following the treatment of MRTX1133, the expressions of critical genes associated with the Wnt signaling pathway were increased in mouse ductal cells, including *CTNNB1* (Fig. 5A and B). The expression levels of *CTNNB1* in mouse ductal cells exhibited a significantly positive correlation with *MGST1* expression (Fig. 5C). Activation of Wnt/β-catenin signaling plays an essential role in the resistance of malignancies to chemoresistance, such as glioblastoma (Huang et al. 2020). It was reported that Wnt/β-catenin signaling confers ferroptosis resistance by targeting GPX4 in gastric cancer (Wang et al. 2022a). However, the expression levels of GPX4 showed no difference between AsPC1 cells and AsPC1-MR cells. The potential role of Wnt/β-catenin signaling in regulating MGST1 expression during the development of resistance to *KARS*^G12D^ inhibitor MRTX 1133 remains unexplored.

Compared with AsPC1 cells, AsPC1 treated with MRTX1133 and AsPC1-MR cells both exhibited activation of the Wnt/β-catenin signaling pathway (Fig. 5D and E). Several critical genes of this pathway, including *NOTCH1*, *CTNNB1*, and *AXIN2*, were significantly increased in AsPC1-MR cells (Fig. 5F). β-catenin, encoded by the CTNNB1 gene, plays a crucial role as a transcriptional activator in the canonical Wnt/β-catenin signaling pathway. We analyzed the active β-catenin levels between the MRTX1133-resistant PDAC cells and parental PDAC cells. The protein levels of active β-catenin were significantly increased in the MRTX1133-resistant PDAC cells (Fig. 5G). Immunofluorescence analysis showed that increased nuclear localization of β-catenin was observed in MRTX1133-treated PDAC cells and MRTX1133-resistant PDAC cells compared to parental cells (Fig. 5H and S3C). Knockdown of CTNNB1 or TCF4 in MRTX1133-resistant PDAC cells significantly decreased MGST1 expression (Fig. 5I and S3D). We also observed that CTNNB1 or TCF4 knockdown decreased the viability of AsPC1-MR and KPC210-MR cells treated with Erastin or MRTX1133 (Fig. 5J and S3E).

Furthermore, we performed a Chromatin Immunoprecipitation (ChIP) assay to examine the impact of the β-catenin/Tcf4 complex on the transcription of *MGST1* transcription in AsPC1, AsPC1 treated with MRTX1133, and AsPC1-MR cells. The putative motif combined with β-catenin/Tcf4 complex in the promoter region of *MGST1* was identified as TTATTTATCT, which was analyzed using GTRD data (Zhu et al. 2021). These results indicated that the putative motif was more prominently detected in the DNA that was precipitated after the addition of the antibodies targeting β-catenin or Tcf4 in AsPC1-MR cells (Fig. 5K). Overall, our results showed that the MRTX1133-resistant PDAC cells exhibited increased nuclear localization and higher protein levels of active β-catenin compared to the parental PDAC cells, thus contributed to MGST1-mediated ferroptosis inhibition.

### PKF-118-310 synergize with MRTX1133 induced ferroptosis in MRTX1133-resistant PDAC cells

**Fig. 6:**
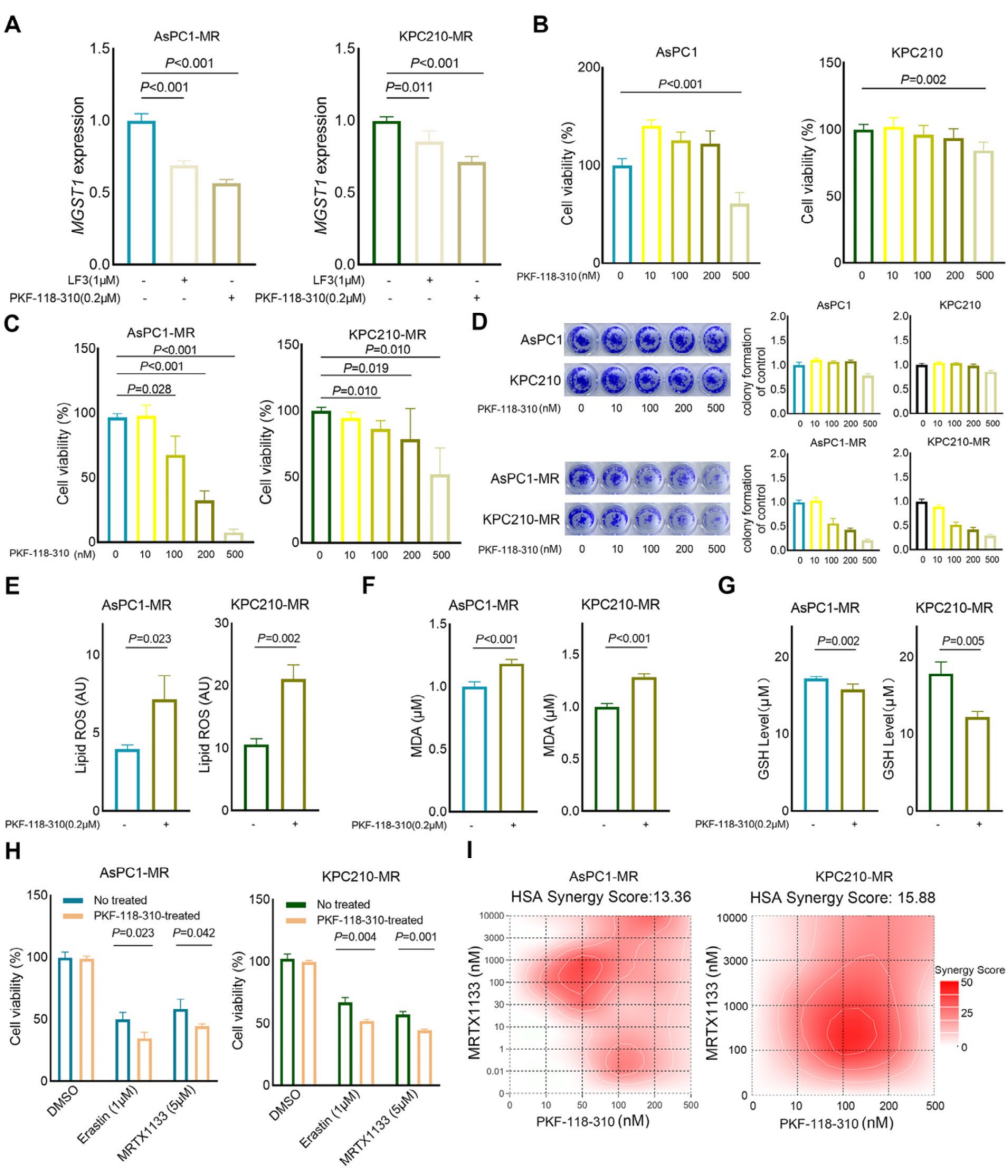
PKF-118-310 synergize MRTX1133 induced MRTX1133-resistant PDAC cells ferroptosis. (**A**) The MGST1 expression levels in MRTX1133-resistant PDAC cells treated with 1 µM LF3 or 0.2 µM PKF-118-310 for 48 h were measured by RT-qPCR detection. (**B**) The cell viabilities of parental PDAC cells treated with different concentration of PKF-118-310 (10 nM, 100 nM, 200 nM, and 500 nM) for 48 h. (**C**) The cell viabilities of MRTX1133-resistant PDAC cells treated with different concentration of PKF-118-310 (10 nM, 100 nM, 200 nM, and 500 nM) for 48 h. (**D**) The cell proliferation after different concentration of PKF-118-310 treatment for 48 h were assessed by colony-formation assay. (**E**) The lipid peroxidation was assessed by BODIPY 581/591 C11 probe using a flow cytometer after the 0.2 µM PKF-118-310 treatment for 48 h. (**F**) The intracellular MDA level was assessed after the 0.2 µM PKF-118-310 treatment for 48 h. (**G**) The intracellular GSH level was assessed after the 0.2 µM PKF-118-310 treatment for 48 h. (**H**) The effects of PKF-118-310 on the cell viabilities after Erastin or MRTX1133 treatment were assessed by CCK8 assay. Pretreatment with 0.2 µM PKF-118-310 for 4 h and the other indicated drugs was carried out for 48 h. (**I**) The synergistic effects of MRTX1133 and PKF-118-310 were measured in MRTX1133-resistant PDAC cells. Data represent means ± SEM from three independent experiments. *P* values were calculated by an unpaired student *t* test or the Mann–Whitney test. ROS: Reactive oxygen species; MDA: malondialdehyde; GSH: Glutathione

Currently, there are no medications or inhibitors that effectively block the expression of MGST1. In order to explore the anti-tumor effect of MGST1 knockdown, we utilized LF3 and PKF-118-310, two antagonist of the β-catenin/Tcf4 complex that possesses anti-tumor properties (Uppada et al. 2018; Fang et al. 2016). Treatment with 1 µM LF3 or 0.2 µM PKF-118-310, both AsPC1-MR cells and KPC210-MR cells showed a significant reduction in *MGST1* expression (Fig. 6A). We thus further investigated the effect of PKF-118-310 on parental PDAC cells and MRTX1133-resistant PDAC cells. PKF-118-310 did not enhanced the acute cytotoxicity in AsPC1 cells and KPC210 cells (Fig. 6B). However, PKF-118-310 effectively induced cell death in AsPC1-MR cells and KPC210-MR cells (Fig. 6C). Colony formation assays also indicated AsPC1-MR and KPC210-MR cells were more resistant to PKF-118-310 (Fig. 6D). Treatment with 0.2 µM PKF-118-310 increased lipid ROS levels and MDA accumulation and caused GSH depletion in MRTX1133-resistant PDAC cells compared with untreated cells (Fig. 6E and G), which resulting in increased sensitivity to Erastin and MRTX1133 (Fig. 6H). These results showed that PKF-118-310 has the ability to repress the MGST1 expression and induce ferroptosis in MRTX1133-resistant PDAC cells.

We next sought to ascertain whether there exists a combinational effect of MRTX1133 with PKF-118-310. The CCK8 experiment was conducted using different concentrations of MRTX1133 (ranging from 0 to 10 µM) and PKF-118-310 (ranging from 0 to 500 nM) in MRTX1133-resistant PDAC cells. The construction of the dose-response matrix data was performed using the SynergyFinder tool. Drug combinations with scores exceeding 10 are considered to exhibit a synergistic effect (Ianevski et al. 2017). The results showed that the combination of MRTX1133 and PKF-118-310 had a synergistic effect in MRTX1133-resistant PDAC cells, as indicated by HSA synergy scores of 13.36 in AsPC1-MR cells and 15.88 in KPC210-MR cells (Fig. 6I). Also, compared with the PDAC tumor cells treated with MRTX1133 or PKF-118-310 alone, combination of the two drugs significantly reduced cell viability (Fig. S4A). These results indicated that PKF-118-310 combination with MRTX1133 synergistically sensitize MRTX1133-resistant PDAC cells to MRTX1133 and reverse MRTX1133 resistance by inducing ferroptosis.

### Combination of PKF-118-310 with MRTX1133 impedes the growth of MRTX1133-resistant PDAC tumors in nude mice

**Fig. 7:**
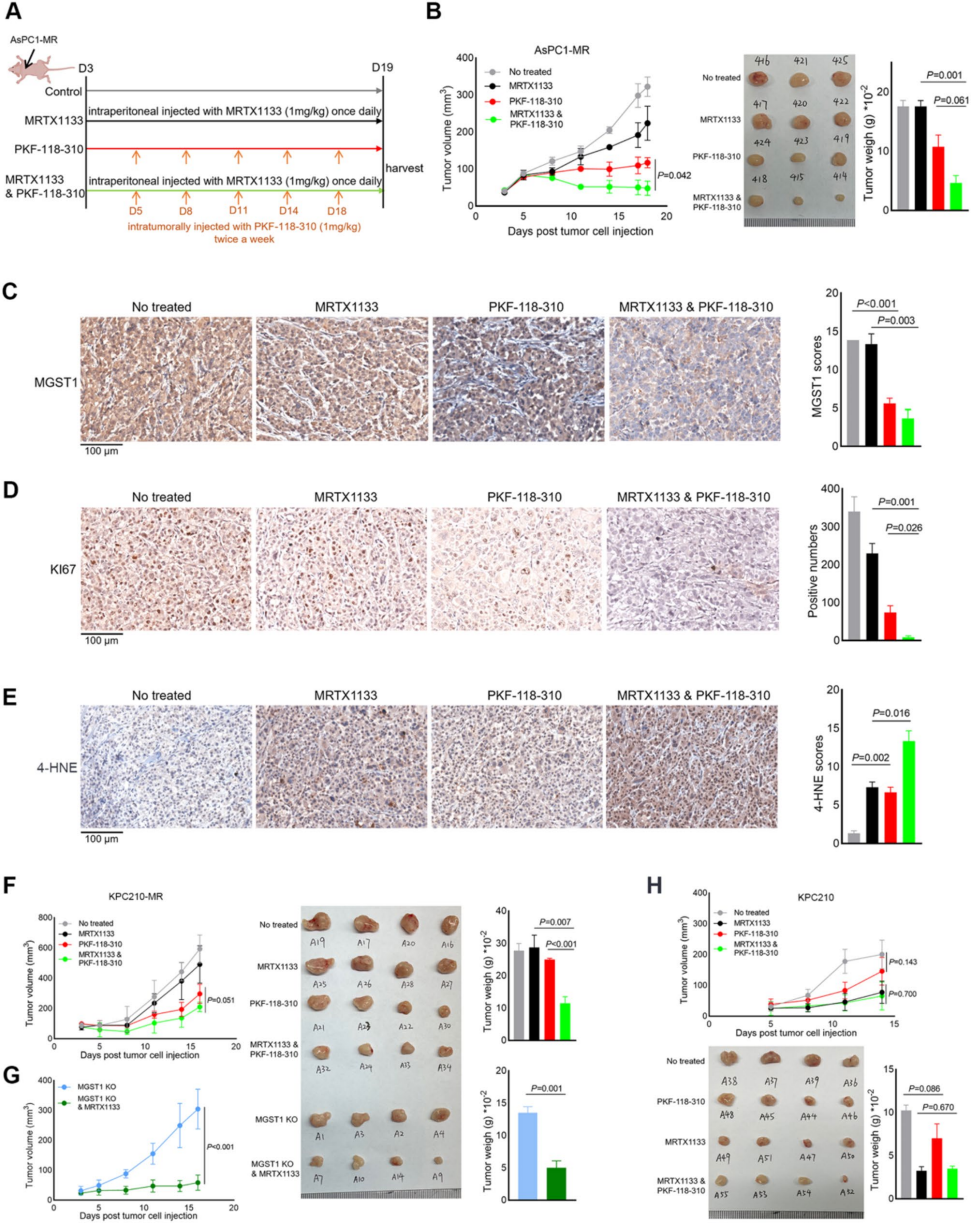
Combination of PKF-118-310 with MRTX1133 restrains the growth of MRTX1133-resistant PDAC tumors in nude mice. (**A**) Experimental scheme. The nude mice were subcutaneously implanted with 1 × 10^6^ AsPC1-MR cells. On the day 3 following subcutaneous, mice were administrated with 1 mg/Kg MRTX1133 through intraperitoneal injection once every day, 1 mg/Kg PKF-118-310 through intratumoral injection once every three days, or combined treatment. Control mice (No treated) were administered vehicle at the same time points. Each group had three mice, which were sacrificed on Day 19. (**B**) The tumour size were measured in mice of four groups every 2–3 days. Images are the removed tumors in the mice that were killed on day 19. Scatter plot shows the tumor weights (means ± SEM). (**C**) Representative images of MGST1 detected in the removed tumor tissues by immunohistochemistry staining of four groups. Scale bar: 100 μm. The graphs showed the average scores of MGST1 calculated from the group of mice (*n* = 3). (**D**) Representative images of KI67 detected in the removed tumor tissues by immunohistochemistry staining of four groups. Scale bar: 100 μm. The graphs showed the positive KI67 cell numbers in each group. (**E**) Representative images of 4-HNE detected in the removed tumor tissues by immunohistochemistry staining of four groups. Scale bar: 100 μm. The graphs showed the average scores of 4-HNE calculated from the group of mice (*n* = 3). (**F**) The nude mice were subcutaneously implanted with 3 × 10^5^ KPC210-MR cells. When a tumor mass formed on Day 3, mice were administrated with 1 mg/Kg MRTX1133 through intraperitoneal injection once every day, 1 mg/Kg PKF-118-310 through intratumoral injection once every three days, or combined treatment. Control mice (No treated) were administered vehicle at the same time points. Each group had four mice. Images are the removed tumors in the mice that were killed on day 16. Scatter plot shows the tumor weights (means ± SEM). (**G**) The nude mice were subcutaneously implanted with 3 × 10^5^ KPC210-MR cells subjected to knockout of MGST1. When a tumor mass formed on Day 3, mice were administrated with 1 mg/Kg MRTX1133 through intraperitoneal injection once every day. Control mice (MGST1 KO) were administered vehicle at the same time points. Each group had four mice. Images are the removed tumors in the mice that were killed on day 16. Scatter plot shows the tumor weights (means ± SEM). (**H**) The nude mice were subcutaneously implanted with 3 × 10^5^ KPC210 cells. When a tumor mass formed at Day 5, mice were administrated with 3 mg/Kg MRTX1133 through intraperitoneal injection once every day, 1 mg/Kg PKF-118-310 through intratumoral injection once every three days, or combined treatment. Control mice (No treated) were administered vehicle at the same time points. Each group had four mice. Images are the removed tumors in the mice that were killed on day 14. Scatter plot shows the tumor weights (means ± SEM). Data represent means ± SEM. *P* values were calculated by an unpaired student *t* test

To further examine the effect of PKF-118-310 on MRTX1133-resistant tumors in vivo, we subcutaneously injected 1 × 10^6^ AsPC1-MR cells into nude mice to induce tumor formation. Once the xenografts were established, the mice were treated with 1 mg/kg MRTX1133 intraperitoneally once daily (*n* = 3), 1 mg/kg PKF-118-310 intratumorally twice a week (*n* = 3), a combination of MRTX1133 and PKF-118-310 (*n* = 3), or left untreated as a control group (*n* = 3) (Fig. 7A). PKF-118-310 showed significant efficacy in inhibiting tumour growth and reducing tumour weight compared to the control group. Combination of PKF-118-310 with MRTX1133 obviously suppressed tumor growth compared with treatment with vehicle, PKF-118-310, or MRTX1133 alone as measured by tumor volumes and tumor weights at the endpoint (Fig. 7B). We sampled mouse tumors and examined the protein levels of MGST1 and Ki67 by immunohistochemical staining (IHC). Intratumorally injection of PKF-118-310 led to a notable decrease in the expression levels of MGST1 and KI67 in the mouse tumors (Fig. 7C and D). To corroborate decreased MGST1 expression conferred the susceptibility of cancer to ferroptosis, we also performed IHC with antibody specific for 4-HNE (a lipid peroxidation marker) to examine the ferroptosis level in xenografts. Intratumorally injection of PKF-118-310 significantly increased the expression of 4-HNE as compared with mice in the control group (Fig. 7E).

In nude mice injected with 3 × 10^5^ KPC210-MR cells subcutaneously, administration of PKF-118-310 synergistically enhanced the effects of MRTX1133 in suppressing tumour growth and reducing tumour weight (Fig. 7F). To better explore the role of MGST1 in MRTX1133-resistant PDAC tumors, we implanted 3 × 10^5^ MGST1 knockout (MGST1 KO) cells subcutaneously into nude mice to induce tumor growth. Treatment with 1 mg/kg MRTX1133 results in significant suppressing of tumour growth and the tumour weight in MGST1 KO group (Fig. 7G). Notably, compared to KPC210-MR cells, MGST1 KO cells exhibited slower tumor growth and enhanced response to MRTX1133 treatment (Fig. S4B). Knockdown of MGST1 in MRTX1133-resistant PDAC cells increased the anti-tumor activity of MRTX1133 in vivo. Additionally, we subcutaneously injected MRTX1133-sensitive KPC210 cells into nude mice, demonstrating tumor inhibition by MRTX1133 alone without observing significantly synergistic effects with PKF-118-310 (Fig. 7H). Altogether, these results revealed that combination of PKF-118-310 and MRTX1133 synergistically inhibited MRTX1133-resistant PDAC tumor growth and induced tumor ferroptosis in vivo.

## Discussion

**Fig. 8:**
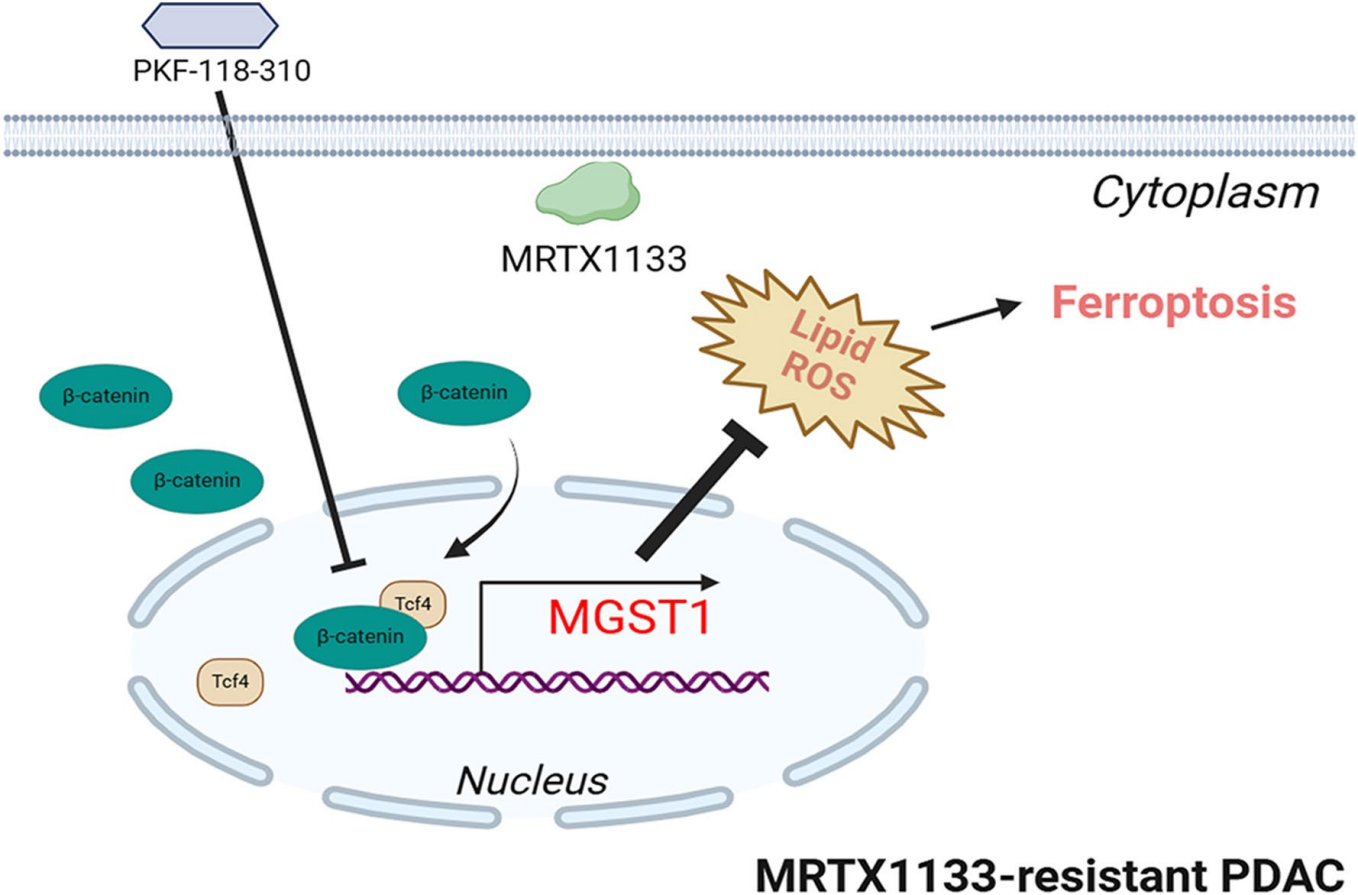
Proposed model of the MGST1 modulated by β-catenin/Tcf4 complex caused ferroptosis inhibition conferred the KRAS^G12D^ inhibitor resistance

Our studies revealed that the MGST1 expression was increased and lipid peroxidation-induced ferroptosis was inhibited in PDAC cells that harbored with the KRAS^G12D^ mutation, when treated with KRAS^G12D^ inhibitor MRTX1133. MGST1 knockdown restores sensitivity to KRAS^G12D^ inhibitor MRTX1133 in the MRTX1133-resistant PDAC cells. Conversely, MGST1 overexpression contributed to ferroptosis inhibition, thus resistant to KRAS^G12D^ inhibitor MRTX1133. When treated with MRTX1133, more increased nuclear localization and higher levels of active β-catenin were observed in MRTX1133-resistant PDAC cells, thus results in higher MGST1 expression. PKF-118-310, a small molecule antagonist of the β-catenin/Tcf4 complex, repressed the *MGST1* expression, and induced MRTX1133-resistant PDAC cells ferroptosis. Administration of PKF-118-310 markedly enhances the therapeutic efficacy of MRTX1133 in both in vitro and in vivo models of MRTX1133-resistant PDAC cells and tumors (Fig. 8). Targeting MGST1 expression could improve MRTX1133 treatment response and inhibit tumor progression, which might represent a promising strategy for PDAC patients with KRAS^G12D^ mutations.

Individuals diagnosed with PDAC have limited effective treatment choices. The mutational activation of the KRAS oncogene is a crucial and necessary stage in initiating and sustaining the progression of PDAC. In mice models, the presence of a KRAS mutation alone led to the development of premalignant pancreatic ductal lesions with 100% penetrance. However, the creation of metastatic pancreatic cancer occurred at a low frequency and was delayed (Hingorani et al. 2003). The major driver mutations of KRAS in PDAC are G12D, which make up around 80–90% (Lennerz and Stenzinger 2015). Hence, directing interventions towards the KRAS^G12D^ mutations holds promise as a potential therapeutic strategy for PDAC. The preclinical studies demonstrated that the KRAS^G12D^ inhibitor MRTX1133 exhibited potent, specific and rapid antitumor activity (Hallin et al. 2022; Kemp et al. 2023; Gulay et al. 2023). Despite the positive outcomes observed in preclinical and clinical studies with novel inhibitors targeting mutant KRAS^G12D^ and its downstream targets, there is a lack of research on understanding resistance mechanisms to these treatments.

The development of resistance to many therapies, including conventional chemotherapy and developing targeted therapy, is a common occurrence in fatal PDAC. Gemcitabine is a first-line chemotherapy drug that has been approved for the management of advanced pancreatic cancer. Nevertheless, gemcitabine did not enhance the overall survival of PDAC patients mainly because of their inherent or acquired resistance to gemcitabine (Conroy et al. 2018), which includes the embolism of glucose, amino acids, and lipids (Qin et al. 2020). Block the activity of mutant KRAS using KRAS inhibitor Sotorasib or MEK inhibitors like trametinib results in the development of adaptive resistance in PDAC. The simultaneous use of inhibitors targeting RAS/MEK and mTORC1/2 leads to long-lasting and effective suppression of PDAC tumour growth, and it also overcomes the development of resistance to the individual drugs (Brown et al. 2020). Furthermore, the development of resistance to KRAS inhibitors in other types of malignancies is also quite feasible. Among a group of 38 patients, consisting of 27 individuals with non-small-cell lung cancer, 10 with colorectal cancer, and 1 with appendiceal cancer, who were administered KRAS^G12C^ inhibitors, it was shown that 45% of the patients developed resistance. The KRAS gene has been found to undergo specific genetic alterations, including G12D/R/V/W, G13D, Q61H, R68S, H95D/Q/R, Y96C, and KRAS^G12C^ allele amplification. These alterations lead to an increase in the quantities of active KRAS protein coupled to GTP and might hinder the effectiveness of drug binding (Awad et al. 2021). The resistance to the KRAS^G12C^ inhibitor Adagrasib is also influenced by changes in numerous RTK-RAS-MAPK pathways. Clinical trials are currently being conducted to investigate the effectiveness of combining RTK or SHP2 inhibitors with mutant KRAS inhibitors (NCT04330664, NCT04185883). These findings suggest that resistance to KRAS inhibitors is a common occurrence. Therefore, it is essential to comprehend the process by which resistance to novel KRAS inhibitors occurs in order to guarantee long-lasting and enduring remissions. According to a recent article, resistance to KRAS inhibitors in vivo is mainly related to other gene mutations in the RAS pathway (Zhao et al. 2021). The limitation of this study was that we only explore the potential mechanisms of expression pattern in this study, we will further study the gene mutations in the MRTX1133-resistant PDAC cells in the future.

It has been reported that KRAS phosphorylates IRE1A in an ERK-dependent manner, which impedes the binding and degradation of IRE1A with HRD1 E3 ligase. While in KRAS inhibitor-resistant tumors, ERK and AKT signaling are overactivated (Lv et al. 2023). Epithelial-to-mesenchymal transition and PI3K-AKT-mTOR signaling are also reported to confer resistance to MRTX1133 therapy in PDAC cell lines and organoid models. The emergence of resistance is accompanied by the amplifications of Kras, Yap1, Myc, and Cdk6/Abcb1a/b, as well as the co-evolution of drug-resistant transcriptional programs (Dilly et al. 2024). Activation of AKT signaling could inhibit the activity of the downstream target protein GSK-3β, promote the accumulation of β-catenin, thus cause the activation of β-catenin pathway (Zhou et al. 2004). In our study, we revealed that the genes associated with the Wnt/β-catenin signaling pathway exhibited increased expression in both MRTX1133-treated and MRTX1133-resistant PDAC cells. Meanwhile, other activated pathway alterations might also contribute to MRTX1133 resistance. In our future study, further research is needed to comprehensively understand all the mechanisms of MRTX1133 resistance.

Increased Wnt/β-catenin signaling pathway activity plays a crucial role in the development of chemoresistance (Huang et al. 2020). A study found that blocking MASTL expression in colorectal cancer cells led to increased sensitivity to 5FU chemotherapy and reduced expression of SURVIVIN and BCL-XL by activating the Wnt/β-catenin signaling pathway (Uppada et al. 2018). It has been documented that the existence of a positive feedback loop between the long non-coding RNA (lncRNA) PVT1 and the Wnt/β-catenin signaling pathway, which played a role in the development of gemcitabine resistance in human pancreatic cancer (Zhou et al. 2020). Pharmacological or genetic suppression of Wnt activity reinstated the responsiveness of cancer cells to chemotherapy, even in mice models (Wickström et al. 2015). In our study, we have discovered the binding element of β-catenin/TCF4 complex in the promoter region of *MGST1*. Upon administration of PKF118-310, MRTX1133-resistant cells exhibited a reduction in MGST1 expression and became more susceptible to MRTX1133. PKF118-310 was reported to exhibit dose-dependent cytotoxicity against HCC cell lines and effectively inhibited tumour growth in a HepG2 xenograft model (Wei et al. 2010). These findings suggested that PKF118-310 could be a potential chemotherapeutic agent for the treatment of PDAC patients who are resistant to KRAS^G12D^ inhibitor MRTX1133.

Due to the release of damage-associated molecular patterns, ferroptosis generally is pro-inflammatory. Knockdown of MGST1 enhanced the efficacy of CD8^+^ T cells in killing B16 melanoma cells in vitro (Zhang et al. 2023). Further investigation is required to determine if the elevated expression levels of MGST1 in KRAS^G12D^ inhibitor-resistant PDAC cells have an impact on the infiltration levels of immune cells.

To summarize, our study demonstrated that KRAS^G12D^ inhibitor MRTX1133 induces PDAC cells to undergo ferroptosis, and MGST1-mediated ferroptosis inhibition lead to resistance of MRTX1133. Mechanismly, the β-catenin/Tcf4 complex played a crucial role in promoting the production of MGST1, which led to prevent ferroptosis in KRAS^G12D^ inhibitor-resistant PDAC in both in vitro and in vivo models. Combined treatment with KRAS^G12D^ inhibitor MRTX1133 and PKF-118-310 exhibited a synergistic effect on KRAS^G12D^ inhibitor-resistant cells and tumours. Therefore, targeting MGST1 expression to modulate ferroptosis might enhance the efficacy of KRAS^G12D^ inhibitor MRTX1133 and inhibit tumor progression, which could be a promising strategy for PDAC patients with KRAS^G12D^ mutations.

## Electronic Supplementary Material

Below is the link to the electronic supplementary material.

Supplementary Material 1

Supplementary Material 2

Supplementary Material 3

Supplementary Material 4

Supplementary Material 5

Supplementary Material 6

Supplementary Material 7

## Data Availability

No datasets were generated or analysed during the current study.
