# Supplementary material for: MGST1 facilitates novel KRASG12D inhibitor resistance in KRASG12D-mutated pancreatic ductal adenocarcinoma by inhibiting ferroptosis
Source: Mol Med. 2024 Nov 5;30:199. doi: 10.1186/s10020-024-00972-y (PMC11536589; doi:10.1186/s10020-024-00972-y)
Supplement: Supplementary file 1 — Supplementary Material 1 [file 10020_2024_972_MOESM1_ESM.docx]

**Supplementary Table 1.** List of quantitative RT-PCR primers.

| Gene | Application |  |  | Orientation | Sequence (5' to 3') |
| --- | --- | --- | --- | --- | --- |
| MGST1 | human |  |  | Forward | ATGACAGAGTAGAACGTGTACGC |
|  |  |  |  | Reverse | TACAGGAGGCCAATTCCAAGA |
| GSTM1 | human |  |  | Forward | TCTGCCCTACTTGATTGATGGG |
|  |  |  |  | Reverse | TCCACACGAATCTTCTCCTCT |
| GPX4 | human |  |  | Forward | GAGGCAAGACCGAAGTAAACTAC |
|  |  |  |  | Reverse | CCGAACTGGTTACACGGGAA |
| CTNNB1 | human |  |  | Forward | AAAGCGGCTGTTAGTCACTGG |
|  |  |  |  | Reverse | CGAGTCATTGCATACTGTCCAT |
| TCF4 | human |  |  | Forward | CAAGCACTGCCGACTACAATA |
|  |  |  |  | Reverse | CCAGGCTGATTCATCCCACTG |
| GAPDH | human |  |  | Forward | GGAGCGAGATCCCTCCAAAAT |
|  |  |  |  | Reverse | GGCTGTTGTCATACTTCTCATGG |
| Mgst1 | mouse |  |  | Forward | CTCAGGCAGCTCATGGACAAT |
|  |  |  |  | Reverse | GTTATCCTCTGGAATGCGGTC |
| Ctnnb1 | mouse |  |  | Forward | ATGGAGCCGGACAGAAAAGC |
|  |  |  |  | Reverse | CTTGCCACTCAGGGAAGGA |
| Tcf4 | mouse |  |  | Forward | CGAAAAGTTCCTCCGGGTTTG |
|  |  |  |  | Reverse | CGTAGCCGGGCTGATTCAT |
| Gapdh | mouse |  |  | Forward | AGGTCGGTGTGAACGGATTTG |
|  |  |  |  | Reverse | TGTAGACCATGTAGTTGAGGTCA |
| MGST1 | Human |  |  | Forward | GGCCTTTGTGTTTACTAGATT |
| （Chip） |  |  |  | Reverse | CAATGTACTTGGTTTGATGCT |
