# Supplementary figures and images for: MGST1 facilitates novel KRASG12D inhibitor resistance in KRASG12D-mutated pancreatic ductal adenocarcinoma by inhibiting ferroptosis
Source: Mol Med. 2024 Nov 5;30:199. doi: 10.1186/s10020-024-00972-y (PMC11536589; doi:10.1186/s10020-024-00972-y)

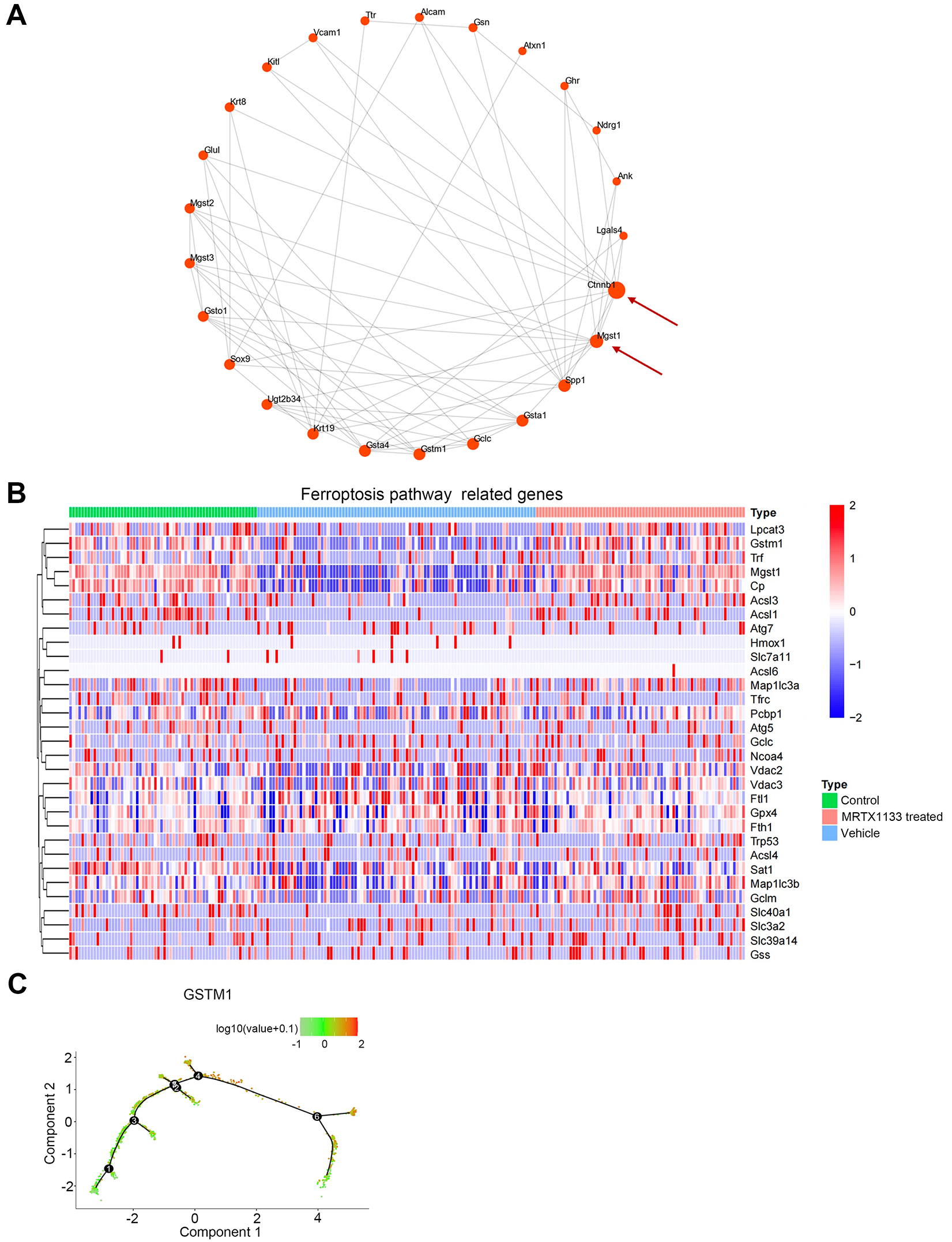

Supplement: Supplementary file 3 — Supplementary Material 3 [file 10020_2024_972_MOESM3_ESM.tif]

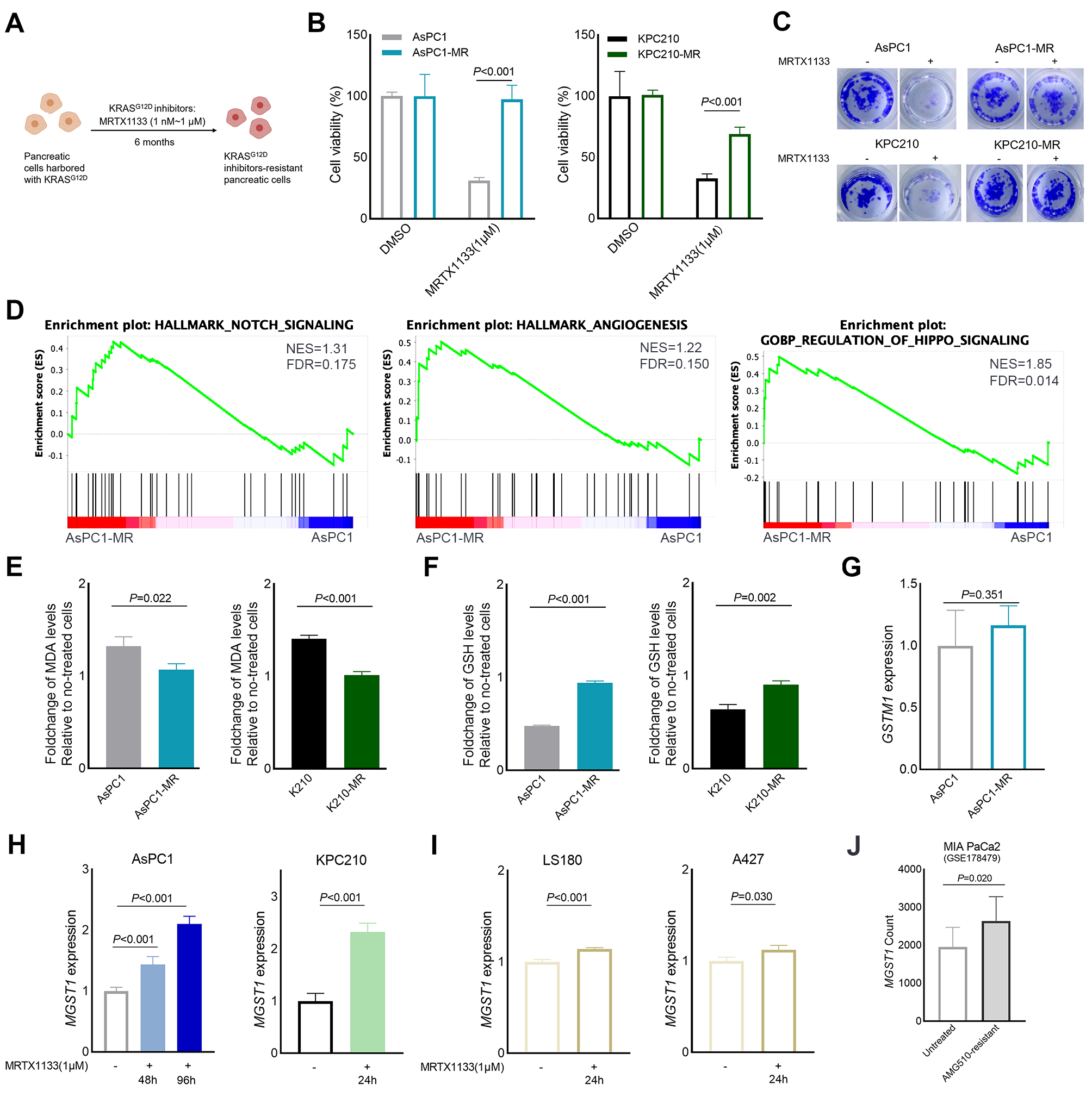

Supplement: Supplementary file 4 — Supplementary Material 4 [file 10020_2024_972_MOESM4_ESM.tif]

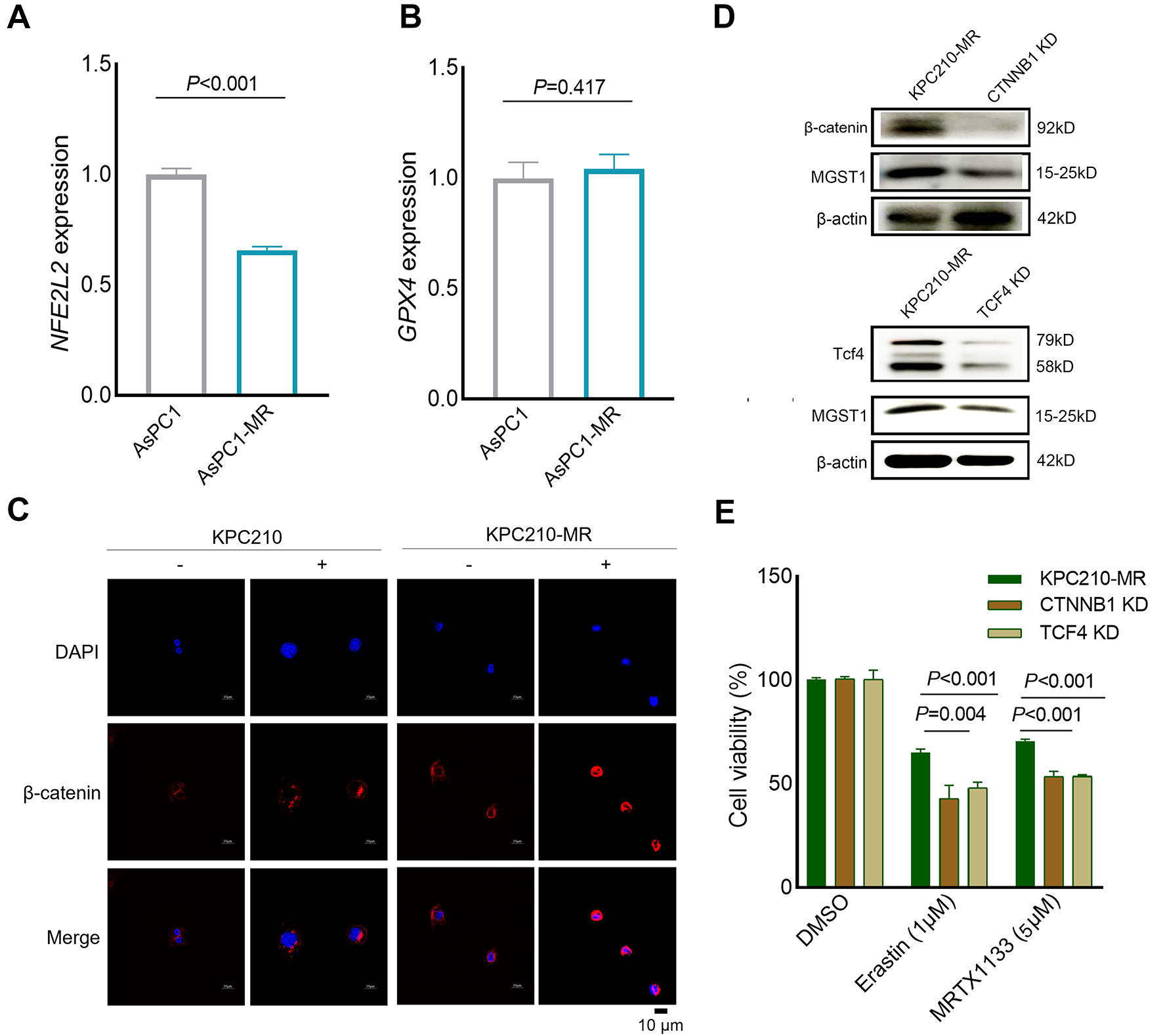

Supplement: Supplementary file 5 — Supplementary Material 5 [file 10020_2024_972_MOESM5_ESM.tif]

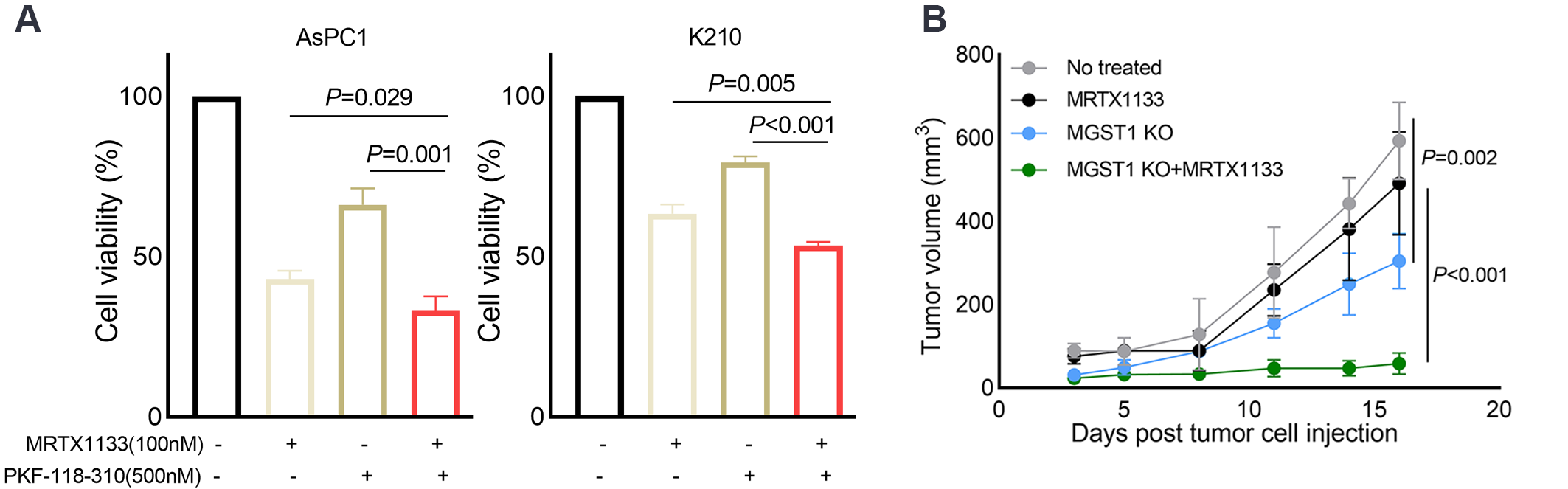

Supplement: Supplementary file 6 — Supplementary Material 6 [file 10020_2024_972_MOESM6_ESM.tif]

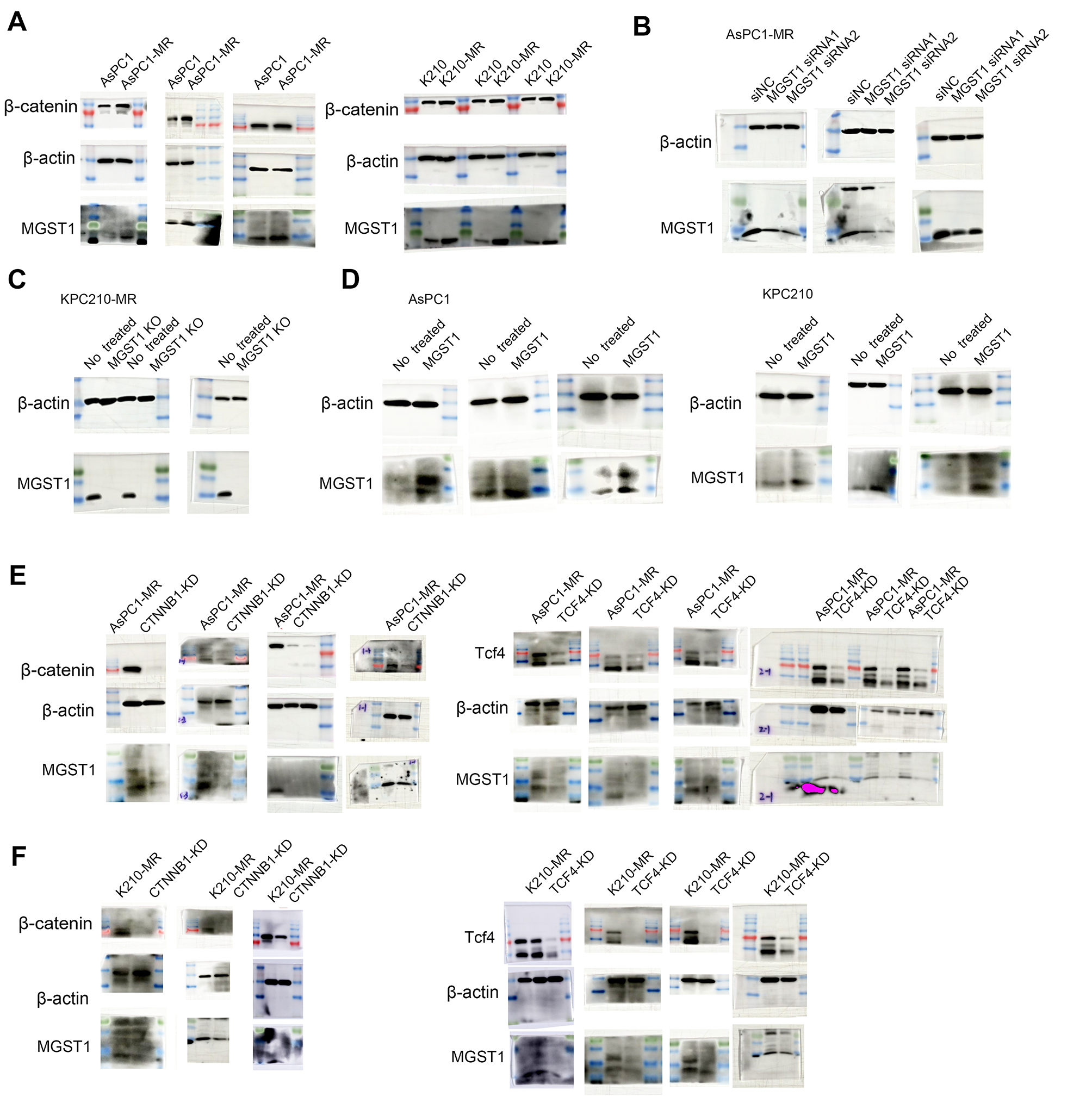

Supplement: Supplementary file 7 — Supplementary Material 7 [file 10020_2024_972_MOESM7_ESM.tif]
